# ATM-kinase deficiency triggers early multi-compartment remodeling of the cerebellar microenvironment

**DOI:** 10.1186/s40478-026-02332-9

**Published:** 2026-05-29

**Authors:** Francesca Montarolo, Luna Berrino, Anita Maria Rominto, Matilde Loddo, Ilaria Balbo, Anastasia Ricci, Giulia Pia Servetto, Ginevra Mango, Kenta Yamamoto, Roberta Parolisi, Antonio Bertolotto, Ruggero Vigliaturo, Michele Menotta, Shan Zha, Filippo Tempia, Eriola Hoxha

**Affiliations:** 1 Department of Neuroscience, University of Torino, Via Cherasco 15, 10125 Torino, Italy; 2 Neuroscience Institute Cavalieri Ottolenghi (NICO), Regione Gonzole 10, 10043 Orbassano, TO Italy; 3 Department of Neurology, College of Physicians and Surgeons, Columbia University, 10032 New York, NY USA; 4 Initiative for Columbia Ataxia and Tremor, Columbia University, New York, NY 10032 USA; 5 Department of Biomolecular Sciences, University of Urbino “Carlo Bo” Campus Scientifico Enrico Mattei, Via Cà le Suore, 2, 61029 Urbino, PU Italy; 6 Department of Earth Sciences, University of Torino, via Valperga Caluso 35, 10125 Torino, Italy; 7 Pathobiology Ph.D. Program, Vagelos College for Physicians and Surgeons, Columbia University, New York, NY 10032 USA; 8 Interdepartmental Centre for Studies on Asbestos and Other Toxic Particulates “G. Scansetti”, University of Torino, via Pietro Giuria 9, 10125 Torino, Italy; 9 Institute for Cancer Genes, Vagelos College for Physicians and Surgeons, Columbia University, New York, NY 10032 USA

**Keywords:** Ataxia Telangiectasia (A-T), Cerebellar atrophy, Motor deficit, Purkinje cell, Degeneration, Excitability

## Abstract

Ataxia Telangiectasia (A-T) is a neurodegenerative disorder characterized by early onset, cerebellar ataxia and progressive motor decline. The causative gene, *ATM* (A-T Mutated), encodes a Ser/Thr kinase, that belongs to the phosphoinositide 3-kinase-related protein kinase family and is crucial for the response to DNA double-strand breaks. While ATM is classically known for its role in the DNA damage response, increasing evidence points to its critical function in maintaining cellular homeostasis, particularly in the central nervous system (CNS). Yet the mechanisms linking ATM-kinase deficiency to cerebellar circuit dysfunction remain poorly defined. Using a CNS Nestin-Cre-restricted mouse model carrying a kinase-dead *Atm* allele combined with a null allele (*Atm*^*KDF/CNS−KO*^), we integrated proteomics, structural and ultrastructural analyses, electrophysiology, and behavioral testing to understand how the loss of Atm kinase activity influence the cerebellar microenvironment from the earliest stages of the disease. Proteomic profiling revealed alterations across four major pathways in *Atm*^*KDF/CNS−KO*^ mice, such as disorganization of the extracellular matrix (ECM), astrocytosis, downregulation of myelin and oligodendrocyte lineage proteins and changes in neuronal excitability. Histological and electron microscopy analyses confirmed increased ECM deposition, astrocytosis and reduction of myelin content without axonal or oligodendrocyte loss, consistent with impaired myelination at early stages. These microenvironmental changes were associated to Purkinje cell dark cell degeneration and increased intrinsic excitability, demonstrating early circuit dysfunction in the absence of overt neuronal loss. Consistently, the compromised cerebellar output is confirmed by the progressive motor impairment developed by *Atm*^*KDF/CNS−KO*^ mice. Our findings indicate that Atm kinase deficiency disrupts cerebellar homeostasis through interconnected and bidirectional mechanisms involving ECM remodeling, astrocytosis, impaired oligodendrocyte functioning and altered intrinsic excitability, reflecting a network level destabilization of the cerebellar microenvironment.

**Supplementary Information:**

The online version contains supplementary material available at 10.1186/s40478-026-02332-9.

## Introduction

Ataxia Telangiectasia (A-T) is a rare autosomal recessive disorder that is characterized by early onset, progressive cerebellar ataxia, oculocutaneous telangiectasia, immunodeficiency, and increased risk of developing cancer [2]. A-T is caused by biallelic mutations in the *Ataxia Telangiectasia Mutated* (*ATM*) gene (Chr 11q22.3-23.1) [13]. The protein encoded by the *ATM* gene is a 370 kDa Ser/Thr kinase member of the phosphoinositide 3-kinase-related protein kinase (PIKK) family, whose members are stress-responsive kinases. ATM is activated by DNA damage, and phosphorylates its downstream targets that coordinate the DNA damage response and regulates the activation of cell cycle checkpoints in order to preserve genome integrity [15]. Besides these well-known roles, Atm plays an important role in the central nervous system (CNS) by modulating glutamatergic and GABAergic synapses [27, 42, 56], and maintaining proper homeostasis of mitochondria and peroxisomes, acting thus as a sensor for changes in the ROS levels to prevent oxidative damage [8, 63].

Neuropathologically, A-T is characterized by a severe and progressive degeneration of the cerebellar cortex, with a massive loss of Purkinje cells (PCs), granule cells, and a reduction of the molecular layer thickness [14]. While cerebellar cortical degeneration has long been considered the primary substrate of the neurological phenotype, increasing evidence indicates that white matter abnormalities are a consistent and clinically relevant component of A-T pathology. Neuroimaging studies in patients have reported marked cerebellar and white matter atrophy [9, 11, 44–46], along with degeneration of cortical-projecting tracts, including corticomotor, corticospinal, and somatosensory pathways [45, 46]. Consistently, myelin disruption has also been observed in non-human primate model of A-T [58], further supporting the notion that impaired myelin homeostasis may represent a conserved and early feature of the disease.

Despite extensive research, the mechanisms linking ATM kinase deficiency to myelin defects and cerebellar circuit dysfunction remain poorly understood. Importantly, ATM deficiency affects multiple cellular processes across neurons and glial cells, raising the possibility that neurodegeneration in A-T does not arise solely from intrinsic neuronal defects [34]. Instead, accumulating evidence points toward cooperative mechanisms [22, 24, 34, 40, 53], , in which disrupted interactions among neurons, astrocytes, oligodendrocytes, and extracellular matrix (ECM), progressively compromise cerebellar circuit integrity and contribute to the neurological decline characteristic in A-T. Oxidative stress and DNA damage, hallmarks of ATM deficiency, are known to profoundly alter ECM composition and mechanics [19], which in turn regulate glial differentiation, myelin maintenance, and neuronal excitability [23, 32]. However, how these microenvironmental changes integrate with myelin pathology and neuronal dysfunction in A-T remains unresolved.

To investigate how the loss of Atm kinase activity impacts the cerebellar circuitry microenvironment, from the earliest stages of the disease, we employed a Nestin-Cre–restricted mouse model carrying a kinase-dead *Atm* allele in combination with a null allele, resulting in selective loss of Atm kinase activity in the CNS [51, 60]. By combining unbiased proteomic profiling with structural, ultrastructural, electrophysiological, and behavioral analyses, we examined how Atm kinase deficiency reshapes the cerebellar tissue environment, with particular focus on ECM deposition, myelin integrity, glial responses, and PC function from early stages of the disease. Our findings reveal that Atm kinase loss triggers an early, coordinated disruption of white matter homeostasis and cerebellar microenvironment stability, preceding neuronal loss and contributing to circuit dysfunction and motor impairment.

## Materials ad methods

### Animals

All experimental procedures have been carried out at the Neuroscience Institute Cavalieri Ottolenghi (NICO), in accordance with the European Communities Parliament and Council Directives of 24 November 1986 (86/609/EEC) and 22 September 2010 (2010/63/EU), approved by the Ethical Committee of the University of Torino and authorized by the Italian Ministry of Health (authorization number: 466/2021-PR). Mice were housed with a 12 h light/dark cycle and had free access to food/water. Adequate measures were taken to minimize pain and discomfort. Female and male mice showed the same phenotype; hence they were pooled together for all the experimental paradigms.

### Generation of the *Atm*^*KDF*^ allele

The *Atm*^*KDF*^ allele carrying knock-in D2880A/N2885K mutations (corresponding to D2870A/N2875K in human ATM) was generated using a strategy similar to that used for the original KD Atm allele [60]. The D2870A/N2875K double mutation was selected because it has been extensively characterized and shown to support normal ATM protein expression while completely abolishing kinase activity [1, 5]. Briefly, the targeting construct was designed to insert a neomycin resistance (NeoR) cassette, oriented opposite to the endogenous Atm promoter, into intron 57 adjacent to the D2880A/N2885K mutations in exon 58. The NeoR cassette was flanked by a pair of FRT sites. A 3.5-kb 5′ homology arm and a 5.1-kb 3′ homology arm were PCR-amplified using high-fidelity polymerase, cloned into shuttle vectors, and fully sequenced. The 5′ arm was subcloned directly into the pEMC targeting vector [33] in the desired orientation. The D2880A/N2885K mutations were introduced into the 3′ arm by site-directed mutagenesis and confirmed by sequencing prior to subcloning into pEMC. The finalized targeting construct was electroporated into CSL3 ES cells (129 strain), and correctly targeted clones were identified by Southern blot analysis. Initial screening was performed using KpnI and EcoRV double digestion with a 5′ genomic probe (Supplementary Fig. 1B-C), yielding a ~ 13.1-kb germline band and a ~ 4.7-kb targeted band due to the introduction of an additional EcoRV site. Correct targeting was further confirmed using a 3′ probe following KpnI and EcoRV digestion, with expected band sizes of ~ 13.1 kb for the germline allele and ~ 10 kb for the targeted allele (Supplementary Fig. 1B-C). More than 12 independently targeted ES cell clones were identified, eight of which were sequenced across exon 58; three clones were confirmed to carry the correct D2880A/N2885K mutations (Supplementary Fig. 1D). Two validated clones were injected to obtain germline transmission. Resulting *Atm*^+/KDFN^ chimeras were crossed with *Rosa26a*^*FLIP/FLIP*^ mice (Jackson Laboratory, Cat. 003946) [10] to induce FLP-mediated excision of the Neoᴿ cassette, generating the *Atm*^KDF^ allele. In the resulting *Atm*^*+/KDF*^ mice, the kinase-dead ATM protein is expressed from the endogenous Atm promoter. A single FRT site remains in intron 57 and does not affect ATM expression.

### Generation of the *Atm*^*KDF*^ murine model

*Atm*^+/KDF^ mice were crossed with ‘floxed’ Atm alleles (*Atm*^*fl/fl*^) mice, containing loxP sites flanking exons 57–58 of the gene (Jackson Laboratory, U.S.A). To inactivate floxed alleles only in the CNS, the *Atm*^*KDF/fl*^ mice were crossed with Nestin-Cre mice (Cre recombinase expressed from a transgene driven by the Nestin promoter) (Jackson Laboratory, U.S.A). Using these crossings, we have developed *Atm*^*KDF/KO*^ *Nestin CRE* positive mice, which are kinase-dead Atm in one allele and knocked out in the other allele only in Nestin positive cells of the CNS, thus preventing premature mortality from cancer predisposition (*Atm*^*KDF/CNS−KO*^). *Atm*^*KDF/fl*^*Cre* negative were used as control mice.

### Proteomic analysis

2 months old mice of wild type (*n* = 6) and *Atm*^*KDF/CNS−KO*^ (*n* = 6) mice were anesthetized with isoflurane (Isoflurane-Vet, Merial, Italy) and decapitated. The cerebella were removed, frozen in ice-cold isopentane, and stored at -80 °C until use. Cerebella were omogenized by a blender using EasyPep MS Sample Kit (Thermo Scientific Pierce). Peptides were solubilized in 0.1% formic acid, and they were quantified through the quantitative colorimetric peptide assay (Thermo Fisher Scientific). 1.9 micrograms of each sample was injected into an UltiMate 3000 RSLC nano system coupled to the Exploris 240 mass spectrometer (Thermo Fisher Scientific) and resolved by Easy-Spray Pepmap RSLC 18 (2 μm, 75 cm × 75 μm) at a flow rate of 200nL/min with a gradient of phase B (80% acetonitrile/0.1% formic acid, solvent A was 0.1% formic acid in water) from 2% to 40% in 250 min. Then (B) was changed to 95% in 30 min, kept for 5 min, and then the column was re-equilibrated for 15 min. Data was acquired in a positive mode and data dependent manner. For MS1 m/z range was set to 350– 1500 at 120,000 resolution (at m/z 200), AGC target 3e6, and auto maximum injection time. MS2 switch when ions intensity was above 5e3, with m/z range in auto mode, normalized HCD energy 30%, AGC target 7.5e4, and maximum injection time 40ms. The resolution was set to 15,000 at m/z 200 and the internal calibrant for employed in run start mode. Analysis was performed by five technical replicates for each sample. Raw data generated by Xcalibur 4.2 software (Thermo Fisher Scientific) were analyzed using Proteome Discoverer 2.5 (Thermo Fisher Scientific), by using SEQUEST algorithm. carbamidomethylation of Cysteines was considered as fixed modification, while as variable serine, threonine or tyrosine phosphorylation. Also, variables oxidation (M) and deamidation (N, Q) were counted. The false discovery rate was evaluated by a target-decoy strategy in concatenated q-value manner. FDR (strict) was set as 0.01, while FDR (relaxed) was set as 0.05. Differentially expressed master proteins (|log 2 FC| ≥ 0.5, *p* < 0.05) were selected and individually evaluated. Fold changes, adjusted p and accension number were submitted to iPathwayGuide software using Advaita’s proprietary Impact Analysis method (Advaita Corporation Ann Arbor, MI).

### Histological procedures and image analysis

2 months-old animals of both genotypes were anesthetized using a cocktail of zoletil (100 mg/kg body weight) and xylazine (5 mg/kg body weight) via intraperitoneal injection. The mice were intracardially perfused initially with a physiological solution (NaCl 0.9%) and then with 4% paraformaldehyde in 0.12 M phosphate buffer, pH 7.2–7.4. Following perfusion, the brains were removed and stored at 4 °C for 2 h immersed in the same fixative. The brains were then transferred to a cryoprotectant solution made of 30% sucrose in 0.12 M phosphate buffer for few days. For each mouse, the cerebellum was separated from the telencephalon and embedded in optimal cutting temperature compound, frozen in ice-cold isopentane. Samples were stored at -80 °C until sectioning. Cerebella and telencephalons were serially cut by a cryostat in 30 μm-thick sagittal and coronal slices respectively and collected in phosphate buffered saline (PBS). Histological procedures were performed on sagittal cerebellar slices of vermis and/or coronal telencephalic sections including the corpus callosum of *Atm*^*KDF/CNS−KO*^ and wild type mice. At least 3 slices/animal and 3 animals/time point were analyzed. All the measurements were done blind to the mouse genotypes.

*Cresyl Violet Staining (Nissl Staining)* was performed on sagittal cerebellar slices of the vermis and on coronal telencephalic sections as previously described [19], to measure the thickness of layers [18], and slice and white matter area. Images were acquired by means of ZEISS Axioscan 7 microscope slide scanner (Oberkochen, Germany) with a Plan Apochromat 20X/0.8 M27 objective. Quantitative evaluations were performed on images with ImageJ software (http://rsbweb.nih.gov/ij/ index. html). The area of the slice and white matter was obtained by drawing the outline.

*The density of PCs* was measured in anti-calbindin immunostained sections using Neurolucida software (MicroBrightField, Colchester, VT, USA) connected to an E-800 Nikon microscope under a 20x objective. Cerebellar slices were incubated overnight at 4 °C with the polyclonal anti-rabbit calbindin (CB38a, Swant, Switzerland) antibody diluted 1:1000 in PBS with 1% TritonX-100 and 1.5% normal donkey serum. Immunohistochemical reactions were performed by the avidin–biotin–peroxidase method (Vectastain ABC Elite kit; Vector Laboratories, Burlingame, CA, USA) and revealed using 3,3′-diaminobenzidine (3% in Tris–HCl) as chromogen as reported in [36]. After processing, sections were mounted on microscope slides with Neo Mount (1.09016, Merck, Darmstadt, Germany). The density of PCs (expressed as number of PCs/mm) was obtained by drawing the outline of PC layer and marking the position of every labelled cell, using the Neurolucida software (MicroBrightField, Colchester, VT, USA) connected to an E-800 Nikon microscope under a 20x objective.

*Silver nitrate Gallyas staining* to detect myelin was performed on sagittal cerebellar slices of vermis and on coronal telencephalic sections as previously described [12, 41]. Images were acquired by means of ZEISS Axioscan 7 microscope slide scanner (Oberkochen, Germany) with a Plan Apochromat 20X/0.8 M27 objective. Quantitative evaluations were performed on images with ImageJ software (http://rsbweb.nih.gov/ij/ index. html). To quantify the Gallyas staining, was considered the fractioned area, defined as the percentage of positive pixels relative to the total area analyzed.

*To detect astrocytes and oligodendrocytes*, immunofluorescence staining was performed as follows. Cerebellar and/or telencephalic sections were incubated overnight at 4 °C with the following primary antibodies: glial fibrillary acidic protein (GFAP; rabbit polyclonal, Z033429-2, Dako, Agilent, Santa Clara, CA, USA; 1:500, RRID: AB_10013382), SRY-box transcription factor 9 (SOX9; goat, AF3075, R&D system; 1:1000, RRID: AB_3750679), SRY-box transcription factor 10 (SOX10; rabbit polyclonal, HPA068898, Sigma-Aldrich, Darmstadt, Germany; 1:1000, RRID: AB_2686054), anti-adenomatous polyposis coli (APC) clone CC1 (mouse, OP80, Calbiochem; 1:700, RRID: AB_3750680), and neuron-glial antigen 2 (NG2, mouse, N8912, Sigma Aldrich; 1:200, RRID: AB_609907). Antibodies were diluted in PBS containing 1% Triton-X-100 and 1.5% normal donkey serum. Sections were then incubated for 2 h at room temperature with secondary anti-rabbit, -mouse and -goat 647- and Cy3-conjugated antibody (Jackson ImmunoResearch Laboratories, West Grove, PA). 4,6-diamidino-2- phenylindole (DAPI, Fluka, Saint Louis, USA) was used to counterstain cell nuclei. Finally, sections were mounted on microscope slides with Tris-glycerol mounting medium supplemented with 10% Mowiol (Calbiochem, La Jolla, CA). GFAP-immunostained images were acquired by means of ZEISS Axioscan 7 microscope slide scanner (Oberkochen, Germany) with a Plan Apochromat 20X/0.8 M27 objective. To quantify the astroglial reactivity, we assessed the density of SOX9+/GFAP positive cells (number of positive cells per mm^2^) and the expression level of GFAP+ astrocytes in the cerebellar vermis by measuring the GFAP+ fractioned area, defined as the percentage of positive pixels relative to the total area analyzed. Images of SOX10, NG2, and CC1-immunostained white matter regions from the cerebellum and corpus callosum were acquired using a Leica TCS SP5 confocal microscope. Confocal images were captured as z-stacked focal planes through the thickness of the slice (30 μm) at 1-µm optical steps with an oil-immersed Plan-Apochromat 40X/1.25 objective, zoom 1.0, and resolution of 1024/1024 pixels and 100 Hz (1 pixel = 0.38 μm). The density of SOX10+, immature SOX10+/NG2 + and mature SOX10/CC1 oligodendrocytes was calculated as the number of positive cells per mm^2^. Adobe Photoshop 6.0 (Adobe Systems, San Jose, CA, RRID: SCR_014199) was used to assemble the final figure panels.

### High resolution light microscopy and transmission electron microscopy

High resolution light and transmission electron microscopy (TEM) were carried out as reported in [17, 29]. 2 to 4 months-old mice were anaesthetized by intraperitoneal injection with zoletil (100 mg/kg body weight) and xylazine (5 mg/kg body weight) and were perfused intracardially with 0.12 M phosphate buffer, pH 7.2–7.4 followed by 2% paraformaldehyde and 2% glutaraldehyde in phosphate buffer. Brains were post-fixed overnight at 4 °C in the same fixative. Vibratome Sect. (300 μm thick) were cut, and post-fixed with 1% osmium tetroxide for 1 h at 4 °C, then stained with uranyl acetate replacement stain (Electron Microscopy Sciences, USA). After dehydration in ethanol, samples were cleared in propylene oxide and embedded in Araldite (Fluka, Saint Louis, USA). Semithin Sect. (1 μm thick) were obtained using an ultramicrotome (Ultracut UCT, Leica, Wetzlar, Germany), stained with 1% toluidine blue and 2% borate in distilled water, and then observed under a light microscope to ensure accurate localization of the corpus callosum. For each sample, bright-field images of the medial corpus callosum were acquired at 100x magnification a Nikon Eclipse 80i microscope. Axon density was quantified using ImageJ software. Each image was overlaid with a grid (via the Grid tool), and eight non-overlapping regions were randomly selected. Axons were manually counted within each square, and axon density was calculated as the number of axons µm^2^. For each sample, the mean axon density was obtained by averaging values from the eight 8 squares. The obtained ultrathin Sects. (70–100 nm) were examined by TEM and STEM. The G-ratio (inner perimeter/outer perimeter), axon diameter, and myelin thickness were determined by using a JEOL, JEM-1400Flash, (Tokyo, Japan) operated at 80 kV, and equipped with a Mega-View-III digital camera and a Soft-Imaging-System (SIS, Münster, Germany). The recorded images were elaborated and measured by using the ImageJ software [47]. The quantification of G-ratio and axon diameter was performed on at least 50 axons/animal and on 4–5 mice per genotype.

### Protein analysis

Protein analysis was performed as previously described [17]. Mice were anesthetized with isoflurane (Isoflurane-Vet, Merial, Italy) and decapitated. The cerebella were removed, frozen in ice-cold isopentane, and stored at -80 °C until use. Cerebella from wild type and *Atm*^*KDF/CNS−KO*^ mice were resuspended in 20% (w/v) RIPA buffer (25 mM Tris–HCl pH 7.4, 150 mM NaCl, 1 mM EGTA, 1 mM EDTA, 1 mM dithiothreitol, 0.5 mM PMSF, 10 µg/ml Aprotinine, 10 µg/ml Leupeptine, 2 mM sodium orthovanadate) and homogenized with a tissue lyser. The lysates were centrifuged at 10,000 g for 20 min at 4 °C and the supernatant was collected and stored at − 80 °C until use. Twenty micrograms of proteins were separated by using a 4–12% Bis-Tris precast gel (Life Technologies) and transferred onto nitrocellulose membrane (GE‐Healthcare). Membranes were than blocked with 50 g/L (5%) nonfat dry milk (Bio‐Rad) in 50 mM Tris–HCl pH7.4, containing 200 mM NaCl and 0.5 mM Tween‐20 and then incubated overnight at 4 °C with primary antibodies. The following primary antibodies were used: myelin basic protein (MBP) (1:1000, Covance, Cat# SMI‐99P‐500, RRID: AB_10120130), laminin-111 (1:5000, Invitrogen, Cat# PA1-16730, RRID: AB_2133633), tenascin (1:1000, Sigma-Aldrich, Cat# T3413, RRID: AB_477574), and Gapdh (1:1000, Abcam, Cat# ab181602, RRID: AB_2630358). HRP‐conjugated goat anti‐mouse (1:5000, Bio‐Rad, Cat# 170–6516, RRID: AB_11125547) and goat anti‐rabbit (1:5000, Bio‐Rad, 170–6515, RRID: AB_11125142) immunoglobulins, in Tris‐buffered saline Tween containing 20 g/L non‐fat dry milk, were used for detection with Luminata Forte Western substrate (WBLUF0100, Millipore, Darmstadt, Germany). Densitometric values were normalized to gapdh. Images were acquired by Chemidoc (Bio‐Rad) and quantified by ImageLab software (RRID: SCR_014210, Bio‐Rad). The protein extracts were run at least three times to check reproducibility.

### Purkinje cell dark cell degeneration assessment

2 to 4 months-old mice were anaesthetized by intraperitoneal injection with zoletil (100 mg/kg body weight) and xylazine (5 mg/kg body weight). They were subsequently fixed by transcardiac perfusion with 2% paraformaldehyde and 2.5% glutaraldehyde in 0.1 M phosphate buffer (pH 7.3). Sagittal sections of the cerebellum (500 μm thick) were cut using a vibratome and post-fixed by incubation for 2 h in 1% (wt/vol) OsO4 supplemented with 1.5% (wt/vol) potassium ferrocyanide. The sections were then dehydrated in a graded ethanol series (30% to 100%, 5 min per step), followed by two 10-minute passages in propylene oxide and 1 h in a 1:1 mixture of propylene oxide and Epon resin. Finally, the samples were embedded in resin. Semithin Sect. (1 μm thick) were prepared using an ultramicrotome (Ultracut UCT, Leica, Wetzlar, Germany) and stained with 1% toluidine blue and 2% borate in distilled water and mounted onto glass cover slips for morphological assessment and quantitation via standard light microscopy [30]. Dark cell degeneration (DCD) was verified by the appearance of a minimum of two of the following features: cytoplasmic darkening, soma shrinkage, and nuclear darkening/chromatin aggregation [30].

### Electrophysiology

#### Slices preparation

Cerebellar slices were prepared as previously described [20, 35]. The animals were anesthetized with isoflurane (Isoflurane-Vet, Merial, Italy) and decapitated. The cerebellar vermis was removed and transferred to an ice-cold artificial cerebrospinal fluid (ACSF) containing (in mM); 125 NaCl, 2.5 KCl, 2 CaCl_2_, 1 MgCl_2_, 1.25 NaH_2_PO_4_, 26 NaHCO_3_, 20 glucose, which was bubbled with 95% O_2_/5% CO_2_ (pH 7.4). Parasagittal cerebellar slices (200 μm thickness) were obtained using a vibratome (Leica Microsystems GmbH, Wetzlar, Germany) and kept for 1 h at 35 °C and then at 31 °C. Single slices were placed in the recording chamber, which was perfused at a rate of 2–3 ml/min with ACSF bubbled with the 95% O_2_/5% CO_2_. All recordings were performed at 31 ± 1 °C temperature.

#### Electrophysiological recordings

Recordings from PCs were performed by an EPC-10 patch‐clamp amplifier (HEKA Elektronik, Lambrecht/Pfalz, Germany). PCs were held at – 70 mV, and data were filtered at 9.1 kHz and sampled at 20 kHz. For current clamp recordings, patch pipettes were filled with a K-gluconate-based internal solution containing (in mM): 140 K-gluconate, 10 HEPES, 0.5 EGTA, 4 MgCl_2_, 4 Na_2_ATP, 0.4 Na_3_GTP and the pH was adjusted to 7.3 with KOH and filtered at 0.2 μm. Hyperpolarizing (form − 400pA to -100 pA) and depolarizing (from + 100 to + 1000 pA) current steps in increments of 100 pA, each lasting 1000 ms and a step interval of 10 s were delivered to PCs. For cell-attached recordings the pipette was filled with NaCl 0.9% solution filtered at 0.2 μm. Gabazine (SR 95531, 20 µM), DAP5 (50 µM) and NBQX (10 µM) were added to the recording chamber to inhibit the GABAA and ionotropic glutamate receptors of PCs, respectively. Data were analyzed using Axograph software (AxoGraph Scientific, Sydney, Australia) and graphs were designed using Igor Pro (Wavemetrics, Lake Oswego, Oregon, USA). Data have been derived from at least three animals per genotype.

### Drugs

All drugs were purchased from HelloBio (Bristol, UK) and were applied via the chamber perfusion line.

### Motor tests

We performed a series of motor tests to evaluate the presence of motor impairment on *Atm*^*KDF/CNS−KO*^ mice. All the experiments were conducted in the morning and with the same light conditions for all animals.

#### Balance beam test

We performed balance beam test to evaluate balance and motor coordination on 2, 12, and 24 months old *Atm*^*KDF/CNS−KO*^ and wild type mice [18]. We used a metal beam 1 cm wide, and 100 cm long suspended 12 cm above the bench. For 24-months old animals, a wider beam (1.5 cm) was used. The mice had to cross the beam to reach a cage enriched with toys. The day of the test was preceded by three days of habituation to allow the mice to familiarize with the experimental apparatus. On the day of the test, the animals were placed to acclimate in the behavioral room at least 15 min before the experiment. The test consisted of three bar crossings for three consecutive days. We recorded the crossings using a video camera and analyzed offline by an operator blind to the genotype. The performance was assessed by measuring the latency required to cross the beam as well as the number of slips. The data are presented as violin plot (median and 25th to 75th percentiles are shown).

#### Footprint analysis

The footprinting test was used to analyze gait parameters. The test was performed as previously described [18, 20]. We used a transparent Plexiglass walkway (20 cm high, 67 cm long and 4 cm wide) elevated 70 cm from the floor. A digital camera was placed underneath the clear platform and video recordings of the mice walking were collected. The mice had to walk at least for three consecutive steps per crossing. Still-frames from the recordings were extracted and analyzed offline using the ImageJ software in order to obtain data concerning stride length (the distance of the same paw in consecutive steps), width (the distance between the center of the two hind or fore paws), the distance between ipsilateral fore paw (FP) and hind paw (HP) placements and the fore and hind stance.

#### Accelerated rotarod test

We assessed the locomotor function using the accelerated rotarod test as previously described [18]. We tested mice for five consecutive days and then again 5 days later (10th day). This protocol was repeated at different ages, at 2, 12, and 24 months, to evaluate long term retention of the motor improvements. In each day, after 1 min training session at constant speed (4 rpm), mice received three test sessions (with a minute interval between sessions) in which the rod (Mouse Rota-Rod, Ugo Basile Biological Research Apparatus, Comerio, Italy) accelerated continuously from 4 to 65 rpm with an acceleration of 5.5 rpm. The latency to fall off the rod was recorded. The cutoff in this experiment was set to 300 s.

### Statistical analysis

All data are presented as mean ± SEM. The Shapiro-Wilk test was used to check whether the data followed a normal distribution. For the data sets that passed the normality test, comparisons were done with unpaired two tailed Student’s *t*-test or by one-way or two-way analyses of variance (ANOVA), followed by the appropriate *post hoc* correction. Data for which the normality test failed, were compared by the Mann-Whitney u-test or by Kruskal-Wallis test. *P* values lesser than 0.05 was accepted as significant. Correlation analysis between proteomic protein expression levels and motor performance was performed using SPSS (IBM SPSS Statistics, version 7). Spearman’s rank correlation coefficient was applied to identify proteins significantly associated with motor outcomes. Proteins showing significant correlations were further analyzed using STRING for protein–protein interaction network construction and functional enrichment analysis. Network outputs were subsequently redrawn and customized using in-house Python scripts.

## Results

### *Atm*^*KD*^ murine model

**Fig. 1:**
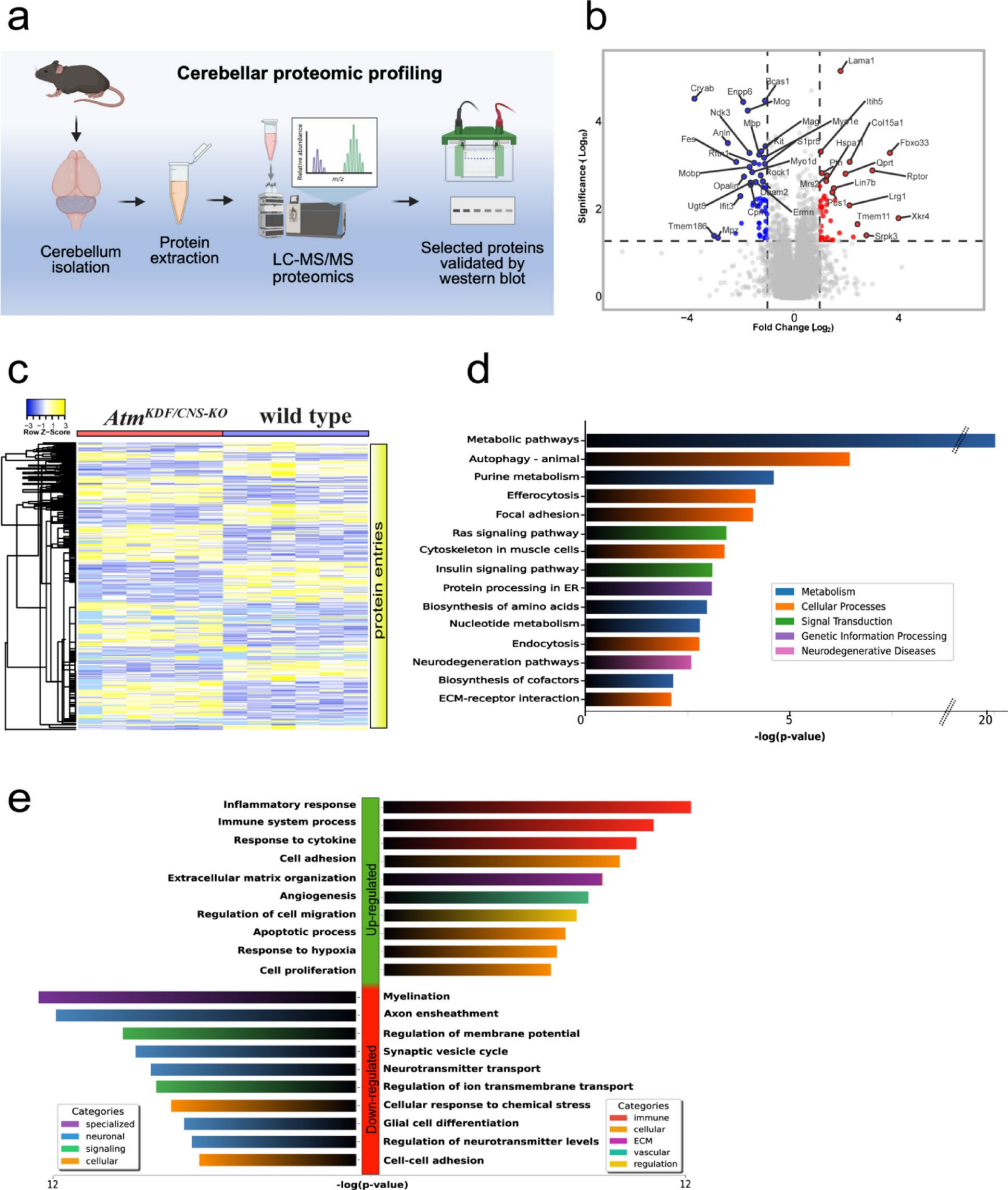
Integration of Quantitative Proteomics and Functional Enrichment in *Atm*^*KDF/CNS−KO*^ mice. Schematic representation of the experiment **(a)**: Differential Expression Profile (Volcano Plot) **(b)**: The plot displays the distribution of differentially expressed proteins (DEPs). Highlighted data points indicate proteins that exceeded the statistical significance (-log10 p-value) and magnitude of change (fold-change) thresholds. Key pathological drivers are labeled to emphasize major biochemical shifts. Hierarchical Clustering Analysis (Heatmap) **(c)**: The heatmap illustrates protein expression patterns across wild type (WT) and *Atm*^*KDF/CNS−KO*^ samples. Hierarchical clustering reveals a clear separation of proteomic signatures, identifying distinct clusters of proteins that are consistently downregulated or upregulated in the disease model. KEGG Pathway Enrichment Analysis **(d)**: Bar graph showing the top enriched pathways based on -log10 p value. Bars are color-coded by functional category. Gene Ontology (GO) Enrichment of Up- and Down-regulated Proteins **(e)**

Our initial *Atm*^*KD*^ allele with knocking in D2880A/N2885K (corresponding to D2870A/N2875K in humans) double-mutation into the conserved catalytic loop also contains a single loxP site after the Cre-mediated deletion of the Neo resistance cassette (NeoR) [60]. Using this KD allele, we successfully demonstrated accelerated oncogenesis using an early and robust Vav-Cre allele, leading to aggressive immature T cell lymphomas [59]. But in the slow progressive B cell lymphoma model or in neuronal models as described here, a rare recombination between the residual loxP site in the KD allele and the loxP sites in the Atm conditional/null allele [62] leads to inactivation (null) of both alleles (Supplementary. Figure 1A) and effectively creates a null model. To avoid this, we have generated a new *Atm*^*KDF*^ allele (termed KDF for FRT), in which the LoxP sites flanked NeoR were replaced by a pair of FRT sites (Supplementary Fig. 1B). Therefore, after the neo-deletion, only an FRT is left (not a loxP) that would not recombine with the loxP on the conditional allele (Supplementary Fig. 1A-B). Southern blot analyses and the Sanger sequencing validate the correct targeting and the presence of the double mutations (Supplementary Fig. 1C-D). Using the Vav-Cre that expresses in all hematopoietic lineages, we confirmed that the newly generated *Atm*^*KDF*^ allele conferred no ATM kinase activity, evidenced by reduced surface TCRβ-positive mature thymocytes and decreased Immunoglobulin class switch recombination at levels comparable to the *Atm*^*−/−*^ and *Atm*^*KD*^ alleles [3, 28, 43, 60] (Supplementary Fig. 1E).

### Differentially expressed proteins in the cerebellum of Atm^*KDF/CNS−KO*^ mice

To identify molecular alterations induced by Atm kinase deficiency, we performed proteomic analysis of whole-cerebellum lysates from *Atm*^*KDF/CNS−KO*^ mice and wild type littermates (Fig. 1a). A total of 8302 master proteins were identified, revealing a profound disruption of cerebellar protein homeostasis in *Atm*^*KDF/CNS−KO*^ mice. Global differential expression analysis demonstrated a widespread molecular phenotype associated with Atm kinase deficiency, as illustrated by the volcano plot (Fig. 1b). We identified 299 differentially expressed proteins: 165 proteins were upregulated, and 134 downregulated in *Atm*^*KDF/CNS−KO*^ mice compared to wild type littermates, with |log2(Fold-change)| ≥0.5 and statistical significance set at *p* < 0.05, revealing a coordinated disruption of multiple cellular compartments (Table S1). Hierarchical clustering analysis (Fig. 1c) based on the Z-score distribution demonstrates a clear-cut proteomic signature that distinguishes *Atm*^*KDF/CNS−KO*^ replicates from the wild type littermates, indicating high reproducibility of the molecular phenotype and supporting the existence of a consistent cerebellar proteomic remodeling induced by Atm deficiency. KEGG pathway enrichment analysis revealed a significant enrichment of cellular processes, metabolic, and signaling pathways (Fig. 1d). Importantly, KEGG enrichment analyses identified ECM-receptor interaction and focal adhesion pathways, indicating early disruptions in the ECM stability and matrix-cell signaling in the cerebellum of *Atm*^*KDF/CNS−KO*^ mice (Fig. 1d). These findings highlight a broad disruption of the cellular homeostasis in the cerebellum of *Atm*^*KDF/CNS−KO*^ mice.

Consistently, Gene Ontology (GO) analysis of the upregulated proteome revealed a strong enrichment of basement membrane and interstitial ECM components together with astrocyte- and immune-related processes (Table S1) (Fig. 1e, top). In contrast, the downregulated proteome was dominated by pathways related to myelination, oligodendrocyte differentiation, axon ensheathment, and neuronal communication (Table S1) (Fig. 1e, bottom), suggesting a coordinated impairment of white matter integrity and axon-glia interactions.

**Fig. 2:**
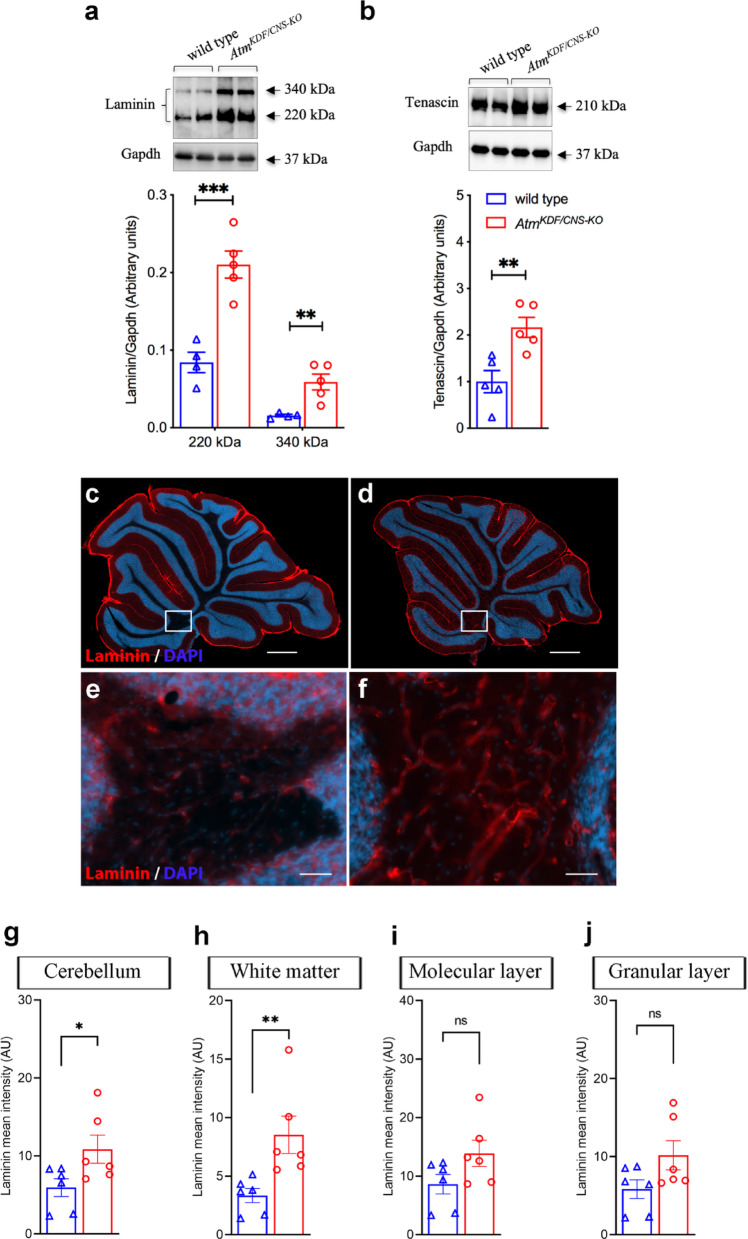
*Atm*^*KDF/CNS−KO*^ mice show increased expression of ECM components. Representative western blots of cerebellar extracts and densitometric quantification for laminin subunits (**a**) and tenascin (**b**) from wild type and *Atm*^*KDF/CNS−KO*^ mice. Gapdh served as loading control. wild type *n* = 4; *Atm*^*KDF/CNS−KO*^ *n* = 5. Representative fluorescent images of the abundance of Laminin+ (red) and Dapi (blue) stained cerebellar sections form wild type (**c**) and *Atm*^*KDF/CNS−KO*^ mice (**d**). Panels (**e**) and (**f**) are the high-resolution confocal images of boxed regions in the upper panels. Scale bars: for (**c**) and (**d**) ; 500 μm; for (**e**) and (**f**) 100 μm. Quantification of Laminin+ mean fluorescence intensity of the signal in total (**g**), white matter (**h**), molecular layer (**i**), and granular layer (**j**) area of the cerebellum. wild type *n* = 6; *Atm*^*KDF/CNS−KO*^ *n* = 6. Statistical analysis was performed using Unpaired Student’s t-test; ***p < 0.001; **p < 0.01; *p < 0.05. Results are reported as mean ± SEM

Based on the proteomic identification of altered ECM proteins, we selected laminin and tenascin for western blot validation because they showed increased abundance (Table S1) and represent functionally distinct ECM components. Laminin is a major component of basement membranes and perivascular extracellular matrix, whereas tenascin is an extracellular matrix glycoprotein involved in tissue remodeling, glial responses and neural plasticity [48]. Western blot analysis confirmed a significant increase in protein levels in *Atm*^*KDF/CNS−KO*^ cerebella for both laminin (220 kDa band: *p* < 0.001 and 340 kDa band: *p* < 0.01, Unpaired Student’s t-test; Fig. 2a) and tenascin (*p* < 0.01, Unpaired Student’s t-test; Fig. 2b), supporting the proteomic evidence of ECM dysregulation in mutant cerebella. Since western blot analysis confirmed increased laminin levels in total cerebellar exctracts, we further assessed the spatial distribution of laminin by immunohistochemistry, to determine if change reflected region-specific accumulation or a more widespread alteration across the cerebellum (Fig. 2c-f). The analysis revealed a significant increase of the laminin intensity in the whole cerebellum (*p* < 0.05, Mann-Whitney test; Fig. 2g), and in white matter (*p* < 0.05, Mann-Whitney test; Fig. 2h). Although no statistically significant difference was found in the molecular (*p* > 0.05, Mann-Whitney test; Fig. 2i) and granular layers (*p* > 0.05, Mann-Whitney test; Fig. 2j), the same trend toward increased expression was observed in *Atm*^*KDF/CNS−KO*^ mice compared to wild type mice.

### *Atm*^*KDF/CNS−KO*^ mice display myelination defects

**Fig. 3:**
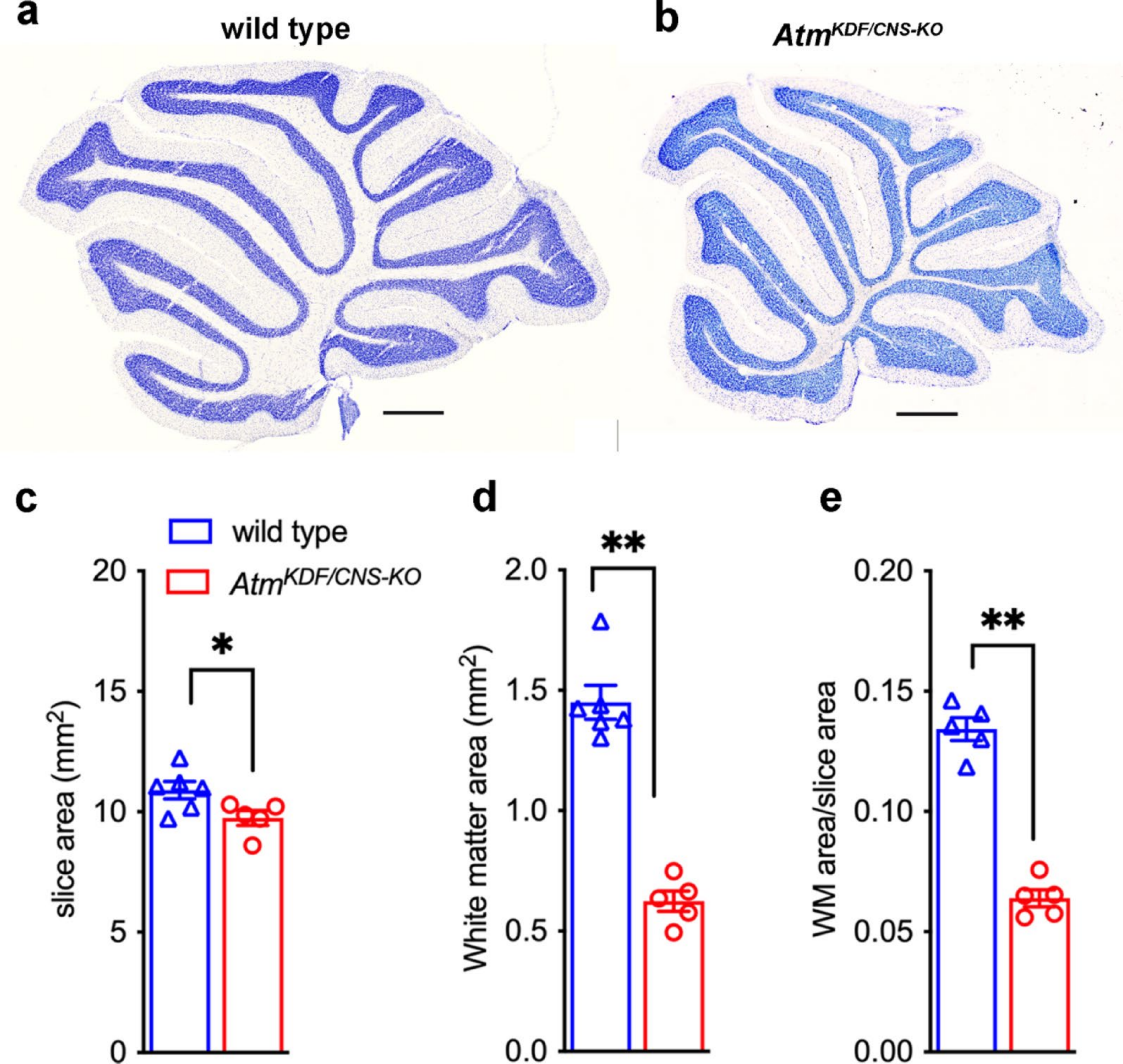
*Atm*^*KDF/CNS−KO*^ mice show white matter area reduction. Nissl-stained sagittal sections of the cerebellum from wild type **(a)** and *Atm*^*KDF/CNS−KO*^ mice **(b)** at 2 months of age (wild type *n* = 6; *Atm*^*KDF/CNS−KO*^ *n* = 5). Significant reduction of the slice area (**c)**, white matter area (**d)**, and of the ratio white matter to slice area (**e)** in the cerebellum of *Atm*^*KDF/CNS−KO*^ mice (*p < 0.05; **p < 0.01, Unpaired Student’s t-test). Results are reported as mean ± SEM. Scale bar 500 μm

**Fig. 4:**
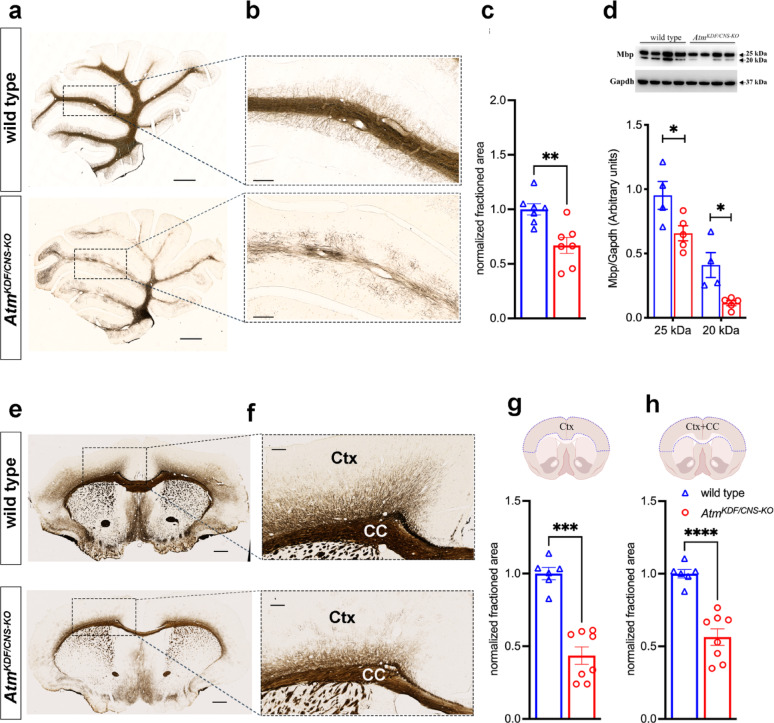
*Atm*^*KDF/CNS−KO*^ mice display myelination defects. Silver nitrate Gallyas myelin staining (brown) in the cerebellum (**a**,** b**) and corpus callosum (**e**,** f**) regions in both *Atm*^*KDF/CNS−KO*^ and wild type mice. Quantification of Gallyas myelin fractioned area in the cerebellar slices from both genotypes **(c)** (wild type *n* = 7; *Atm*^*KDF/CNS−KO*^ *n* = 7). Representative western blots of cerebellar extracts from wild type and *Atm*^*KDF/CNS−KO*^ mice and densitometric quantification of Mbp protein subunits (wild type *n* = 4; *Atm*^*KDF/CNS−KO*^ *n* = 5). Gapdh served as loading control **(d)**. Quantification of Gallyas myelin fractioned area in the dorsal cortex and corpus callosum from both genotypes **(g**,** h)** (wild type *n* = 6; *Atm*^*KDF/CNS−KO*^ *n* = 8). Scale bars: for **(a)** and **(d)** 500 μm; for **(b)** and **(f)** 100 μm. **p < 0.01; ***p < 0.001; ****p < 0.0001, Unpaired Student’s t-test. Results are reported as mean ± SEM. Abbreviations: cc, corpus callosum; ctx: cortex

Given the strong enrichment of ECM, glial and myelin-related pathways, we next asked whether these molecular changes corresponded to structural and ultrastructural alterations. We examined whether the cerebellar white matter was affected in *Atm*^*KDF/CNS−KO*^ mice. Despite the normal foliation, overall cytoarchitecture, and preserved thickness of the molecular and granular layers (Supplementary Fig. 2), *Atm*^*KDF/CNS−KO*^ mice showed a significant reduction in the cerebellar slice area compared to wild type controls (*p* < 0.05, Unpaired Student’s t-test: t_(9)_ = 2.40) (Fig. 3a-c). This reduction was primarily driven by with a significant shrinkage of the cerebellar white matter area (*p* < 0.01, Mann-Whitney test; Fig. 3d). Moreover, the white matter are/slice area ratio was significantly reduced in *Atm*^*KDF/CNS−KO*^ mice (*p* < 0.01, Mann-Whitney test) (Fig. 3e). To directly assess myelin integrity, we performed Gallyas staining on cerebellar sections (Fig. 4a, b). The analysis revealed a marked reduction of myelin staining in *Atm*^*KDF/CNS−KO*^ mice compared to control animal (*p* < 0.01, Unpaired Student’s t-test: t_(12)_ = 3.73; Fig. 4c). In parallel with the structural analyses, we assessed the expression level of Mbp, the most abundantly expressed myelin protein in the CNS, by performing western blotting of cerebellar extracts. Quantitative analyses revealed a significant reduction in both isoforms of Mbp in *Atm*^*KDF/CNS−KO*^ mice compared to wild type littermates (*p* < 0.05, Unpaired Student’s t-test; Fig. 4d), supporting the presence of an early hypomyelinating phenotype.

**Fig. 5:**
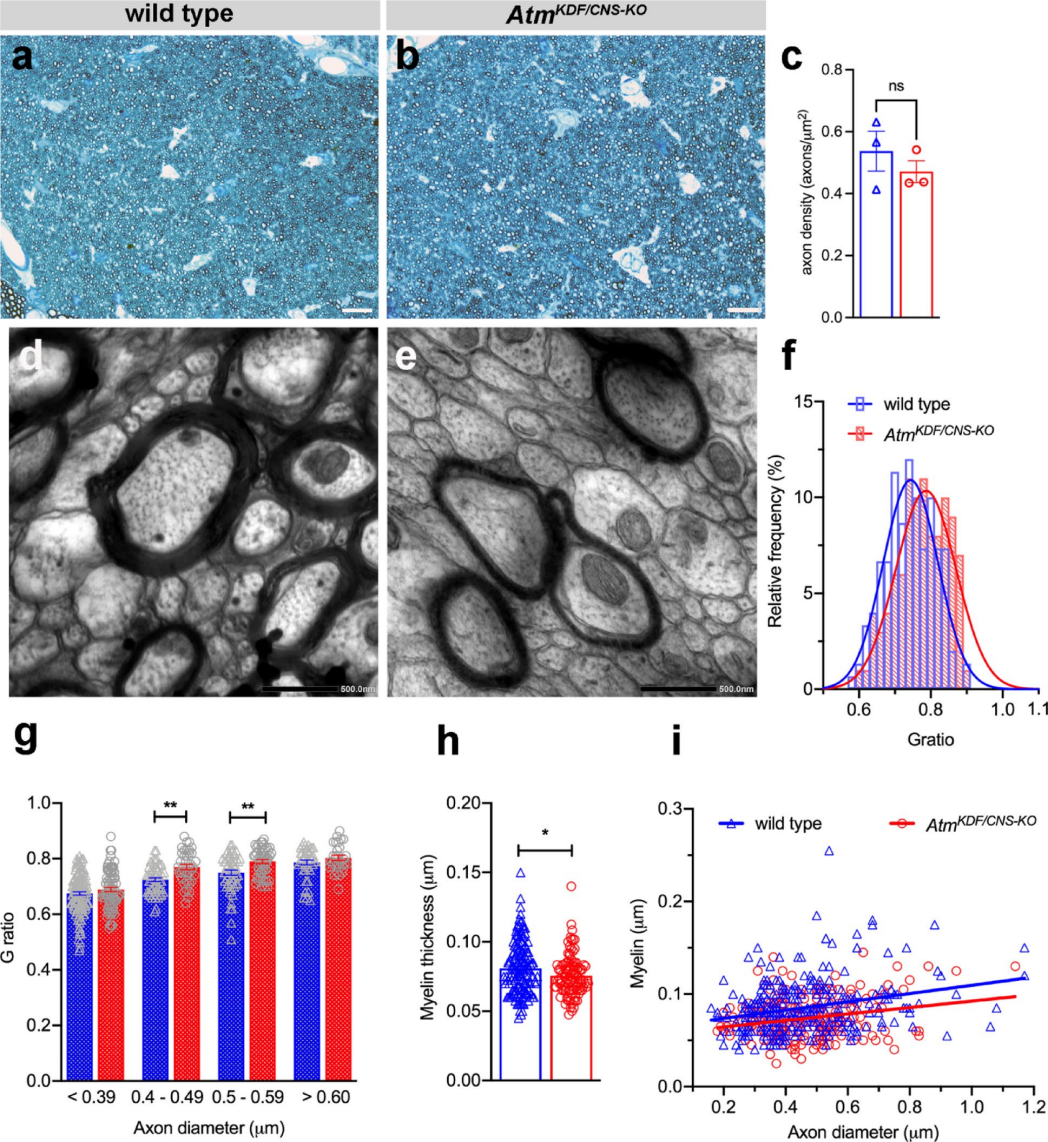
Ultrastructural defects of myelin in *Atm*^*KDF/CNS−KO*^ mice. Representative toluidine blue staining of the corpus callosum on semithin sections of wild type (*n* = 3) (**a**) and *Atm*^*KDF/CNS−KO*^ mice (*n* = 3) (**b**). Scale bar, 20 μm. Quantification of myelinated axon density in toluidine blue staining (**c**). Data are represented as mean ± SEM. Representative transmission electron microscopy image of corpus callosum from wild type (**d**) and *Atm*^*KDF/CNS−KO*^ mice (**e**). Scale bar, 500 nm. *Atm*^*KDF/CNS−KO*^ mice exhibit a significant rightward shift of the cumulative frequency in the g-ratio distribution (*p* < 0.001, Mann-Whitney test) **(f)** and significant higher g-ratios in medium-caliber axons compared to wild type mice **(g)**. Reduced mean myelin thickness in *Atm*^*KDF/CNS−KO*^ mice (*p* < 0.05, Mann-Whitney test) **(h)**. Scatter plot of myelin thickness values across all axon diameters for both genotypes **(i).** Results are reported as mean ± SEM. *p < 0.05, **p < 0.01

To determine whether myelin abnormalities were restricted to the cerebellum, we analyzed the corpus callosum (CC), the largest white matter tract in the brain. *Atm*^*KDF/CNS−KO*^ mice showed a significant reduction in CC thickness compared to wild type littermates (7.56 ± 0.37 vs. 6.67 ± 0.32 μm, respectively, *p* < 0.05, Unpaired Student’s t-test: t_(7)_ = 3.03). Gallyas staining (Fig. 4e, f) revealed a significant reduction of the myelin content within the CC (cortex: *p* < 0.001, Mann-Whitney test; Fig. 4g; cortex+corpus callosum: *p* < 0.0001, Unpaired Student’s t-test: t_(12)_ = 6.17; Fig. 4h), together with a patchy appearance of the myelin in the overlying cortical regions (Fig. 4f), indicating that white matter pathology in *Atm*^*KDF/CNS−KO*^ mice is not confined to the cerebellum. To determine whether reduced myelin staining reflected axonal loss or primary myelin defects, we analyzed semithin and ultrastructural sections of the corpus callosum. Quantification of toluidine blue-stained CC sections revealed comparable axonal densities between genotypes, indicating that Atm kinase deficiency does not affect axon number at this stage (*p* > 0.05, Unpaired Student’s t-test; Fig. 5a-c). TEM revealed several abnormalities in myelin ultrastructure in *Atm*^*KDF/CNS−KO*^ mice (Fig. 5d, e). We evaluated myelin thickness by calculating the g-ratio (myelin thickness relative to axon diameter), defined as the ratio between the inner axonal diameter and the total diameter of the myelinated fiber. Cumulative frequency analysis revealed a significant rightward shift in the g-ratio distribution in *Atm*^*KDF/CNS−KO*^ mice, consistent with thinner myelin sheaths (*p* < 0.001, Mann-Whitney test; Fig. 5f). Two-way ANOVA analysis of g-ratio as a function of axonal diameter showed a significant main effect of genotype (*p* < 0.0001, Two-way ANOVA: genotype effect, F_(1, 472)_ = 21.38; Fig. 5g), with Sidak’s *post hoc* multiple comparisons indicating a selective increase in g-ratio in medium-caliber axons in *Atm*^*KDF/CNS−KO*^ mice compared to wild type littermates (*p* < 0.01; Fig. 5g). Consistent with these findings, direct measurements of myelin thickness confirmed a significant reduction in *Atm*^*KDF/CNS−KO*^ mice (*p* < 0.05, Mann-Whitney test; Fig. 5h). Linear regression analysis of myelin thickness across all axon diameters revealed a significant reduction of the intercept in *Atm*^*KDF/CNS−KO*^ mice (*p* < 0.0001) without a significant change in slope, indicating a global reduction in myelin thickness rather than defective scaling with axon caliber (Fig. 5i). To assess whether the reduction in myelin resulted from oligodendrocyte depletion, we quantified cells expressing the transcription factor SOX10, as a marker of the oligodendrocyte lineage across all stages of their development, immature oligodendrocytes expressing SOX10 and NG2, and mature oligodendrocytes expressing SOX10 and CC1 [21].Notably, the density of SOX10+, SOX10+/NG2+, and SOX10+/CC1 + cells in both the cerebellum and corpus callosum was comparable between genotypes (Supplementary Fig. 3), indicating that myelin defects occur in the absence of oligodendrocyte loss and suggesting impaired myelin maintenance rather than reduced cell number.

### *Atm*^*KDF/CNS−KO*^ mice show cerebellar astroglial reactivity

**Fig. 6:**
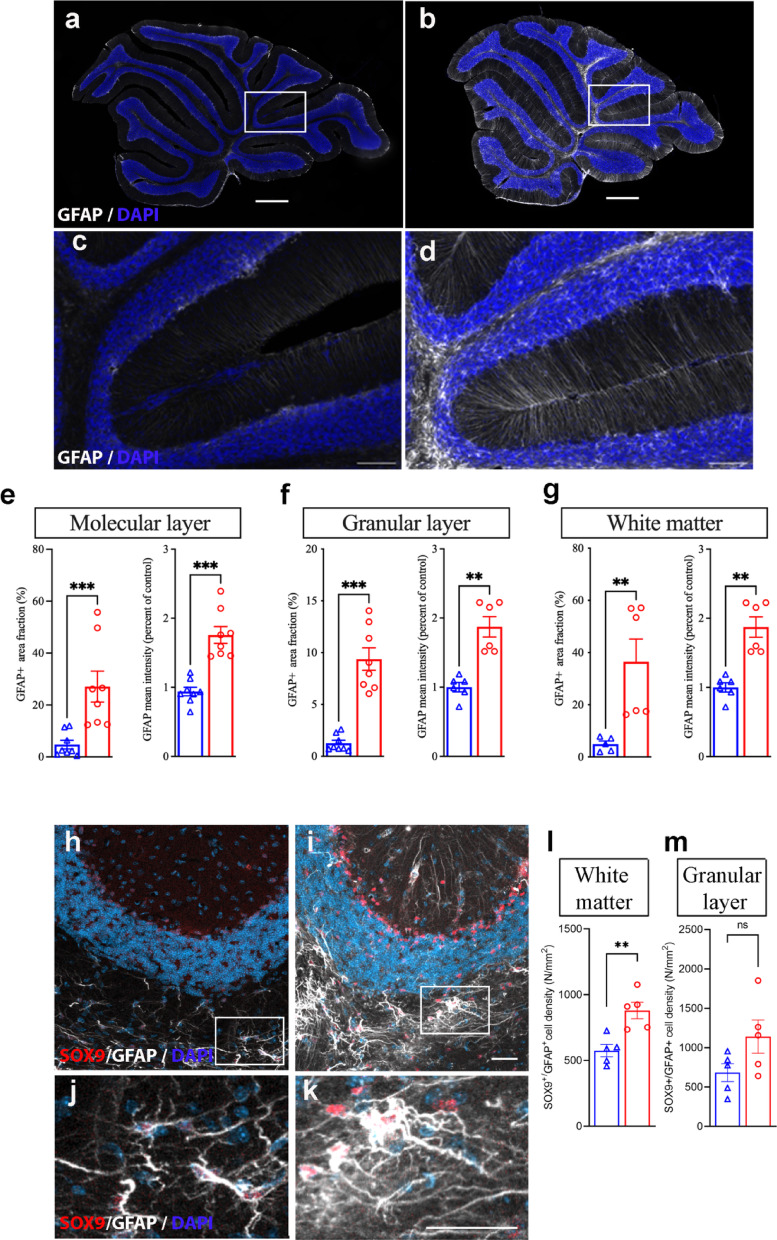
Cerebellar astroglial reactivity in *Atm*^*KDF/CNS−KO*^ mice. Representative fluorescent images of the abundance of GFAP+ (white) astrocytes and Dapi (blue) stained cerebellar sections form wild type (**a**) and *Atm*^*KDF/CNS−KO*^ mice (**b**). Panels (**c**) and (**d**) are the high-resolution confocal images of boxed regions in the upper panels. Quantification of GFAP+ fractioned area and mean fluorescence intensity of the signal in the molecular layer (**e**), granular layer (**f**), and white matter (**g**) area of the cerebellum. Representative fluorescent images of the abundance of SOX9+ (red), GFAP+ (white) astrocytes and Dapi (blue) stained cerebellar sections form wild type (**h**) and *Atm*^*KDF/CNS−KO*^ mice **(i)**. Panels **(j)** and **(k)** are the high-resolution confocal images of boxed regions in the upper panels. Quantification of SOX9+/GFAP+ astrocyte cell density in the white matter (**j**,** l**), and granular layer (**k**,** m**) of the cerebellum. **p < 0.01; ***p < 0.001, (wild type *n* = 8; *Atm*^*KDF/CNS−KO*^ *n* = 8). Unpaired Student’s t-test. Results are reported as mean ± SEM. Scale bars: for (**a**) and (**b**) 500 μm; for (**c**) and (**d**) 100 μm; and for (**h**), (**i**), (**j)**, and (**k**) 50 μm

Given the prominent white matter abnormalities, we next investigated whether Atm kinase deficiency was associated with astroglial activation, a hallmark of disrupted neuron–glia interactions in cerebellar degeneration [6, 61]. Immunofluorescence of cerebellar slices with GFAP, revealed a robust increase in GFAP immunostaining in *Atm*^*KDF/CNS−KO*^ mice across all cerebellar regions examined (Fig. 6a-d). Specifically, the percentage area covered by GFAP was higher in the molecular layer (*p* < 0.001, Mann-Whitney test), granular layer (*p* < 0.001, Mann-Whitney test), and white matter area (*p* < 0.01, Mann-Whitney test). Similarly, GFAP mean intensity was significantly increased in the molecular layer (*p* < 0.001, Mann-Whitney test), granular layer (*p* < 0.01, Unpaired Student’s t-test: t_(14)_ = 8.94), and white matter (*p* < 0.01, Mann-Whitney test) (Fig. 6e-g). To further characterize the astroglial changes suggested by GFAP immunostaining, we quantified SOX9+/GFAP+ cell density in vermal cerebellar slices from both genotypes (Fig. 6h-k). Our analysis revealed a significant increase of density of SOX9+/GFAP+ cells in the cerebellar white matter of *Atm*^*KDF/CNS−KO*^ mice compared to wild type littermates (*p* < 0.01, Mann-Whitney test) (Fig. 6l). No significant differences between genotypes were observed in the granular layer (*p* > 0.05, Mann-Whitney test) (Fig. 6m).

### *Atm*^*KDF/CNS−KO*^ mice show early signs of Purkinje cell degeneration

**Fig. 7:**
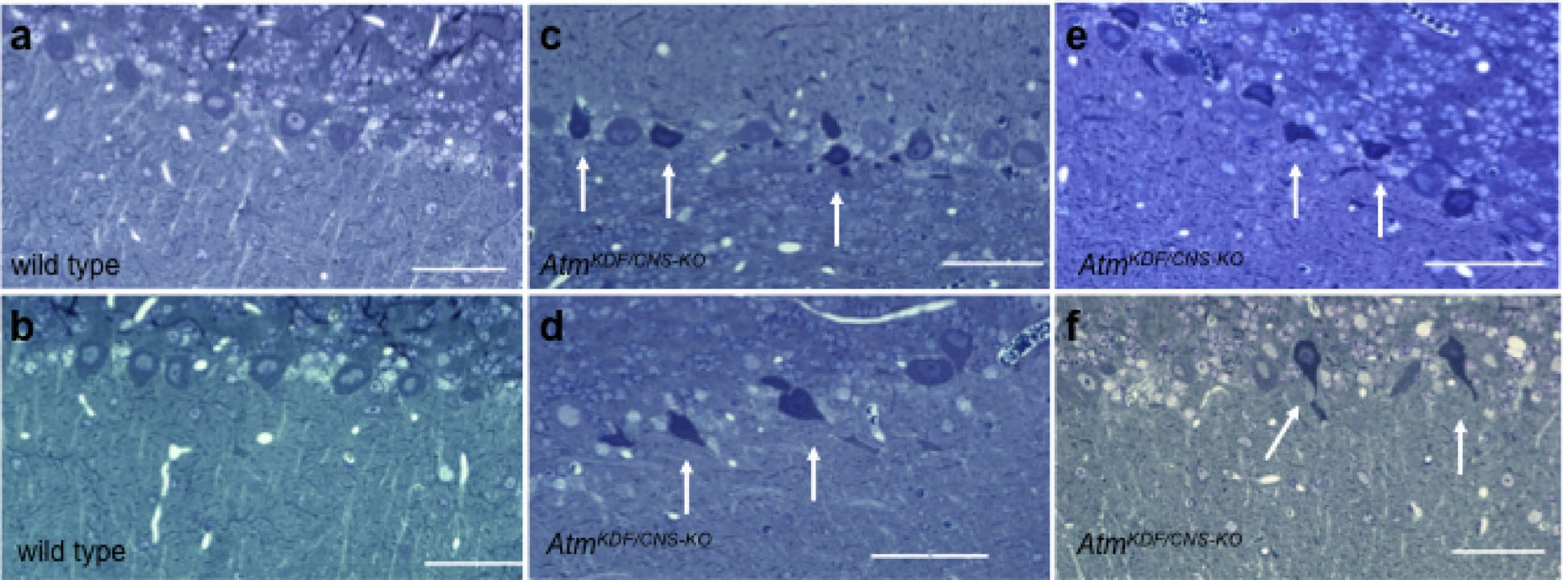
Dark cell degeneration (DCD) of Purkinje cells in *Atm*^*KDF/CNS−KO*^ mice. Toluidine blue staining of sagittal cerebellar sections obtained from wild type (**a**,** b)** and *Atm*^*KDF/CNS−KO*^ **(c**,** e**,** d**,** f)** mice. Arrows indicate DCD positive (darker) cells in the *Atm*^*KDF/CNS−KO*^ which are absent in wild type slices. Scale bar 50 μm

White matter disruption and astrocytosis are known to alter ionic homeostasis and axonal conduction, potentially increasing the vulnerability of neuronal cells [38, 49, 57]. To assess whether PCs were affected at early stages of the disease, we first quantified PC density on calbindin-stained cerebellar sections. At 2 months of age, PC density was comparable between genotypes, indicating the absence of neuronal cell loss at this stage (wild type: 28.69 ± 1.35 cells/mm vs. *Atm*^*KDF/CNS−KO*^: 28.26 ± 1.14 cells/mm, *p* > 0.05, Unpaired Student’s t-test: t_(16)_ = 0.24). We next investigated whether PCs exhibited early degenerative changes by assessing DCD [50]. DCD is a type of neuronal death characterized by a shrunken, electron-dense appearance of the cell body and nucleus [50]. While no DCD PCs were detected in wild type mice (Fig. 7a-b), *Atm*^*KDF/CNS−KO*^ mice displayed multiple PCs with DCD-like morphology (Fig. 7c-f**).** The presence of DCD PCs in the absence of any exogenous insult suggests an increased intrinsic vulnerability of *Atm*^*KDF/CNS−KO*^ PCs to degeneration compared to wild type ones.

### Purkinje cells exhibit early hyperexcitable phenotype in *Atm*^*KDF/CNS−KO*^ mice

**Fig. 8:**
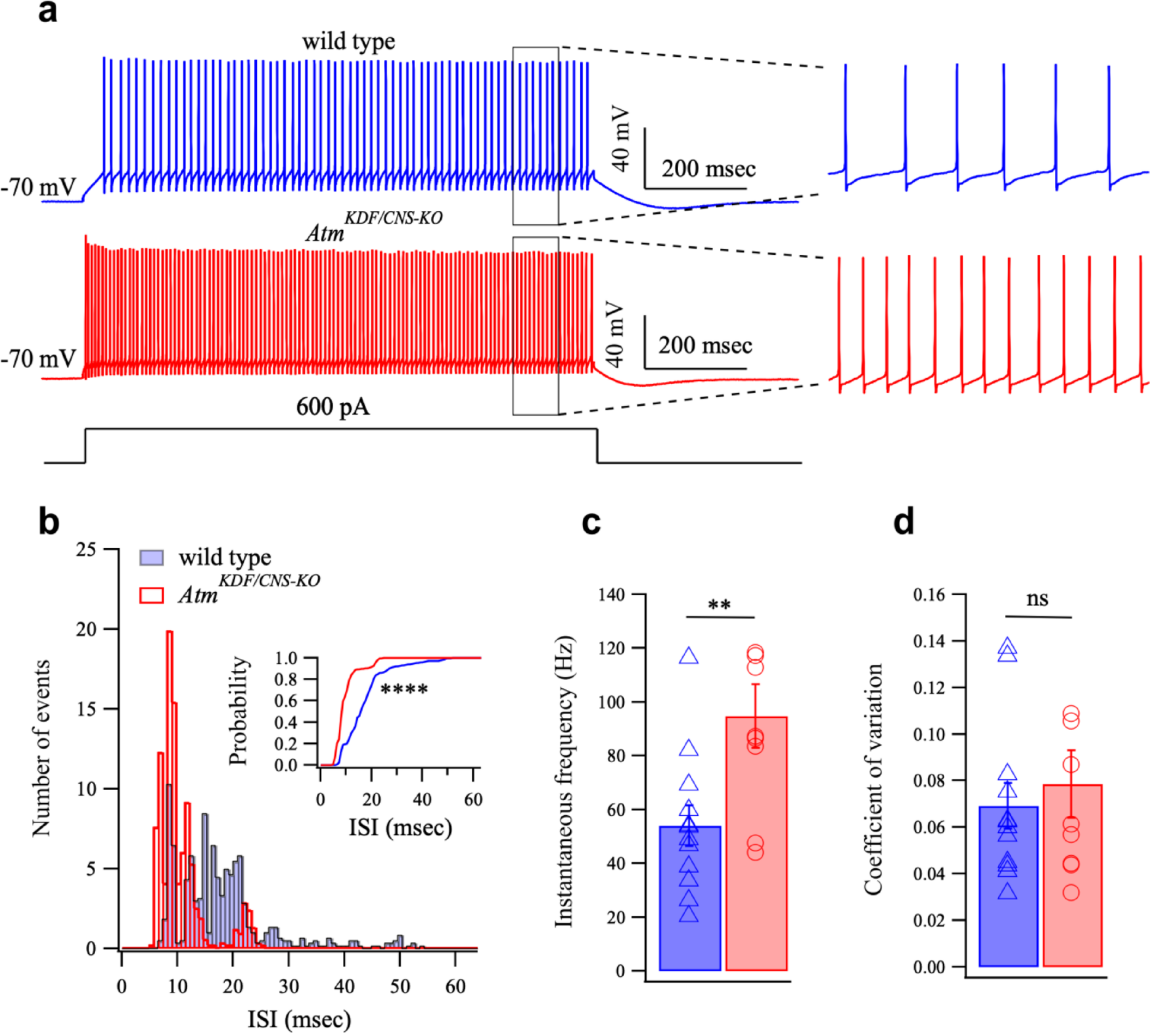
Increased evoked firing frequency of *Atm*^*KDF/CNS−KO*^PCs. Representative traces of action potential discharge evoked by the injection of a 600pA depolarizing step current in wild type (blue trace) and *Atm*^*KDF/CNS−KO*^ PCs (red trace) **(a)**. Distribution histograms of interspike intervals (ISI) from the wild type and *Atm*^*KDF/CNS−KO*^ PCs (**b**) and their cumulative distribution (inset in b) (*p* < 0.0001, K-S test). Increased mean instantaneous frequency in *Atm*^*KDF/CNS−KO*^ PCs (**c**) (*p* < 0.01, Student’s t-test). Mean coefficient of variation of ISI (**d**). *Atm*^*KDF/CNS−KO*^ PCs (cells: *n* = 9); wild type (cells: *n* = 12); **p < 0.01; ****p < 0.0001. Results are reported as mean ± SEM

**Fig. 9:**
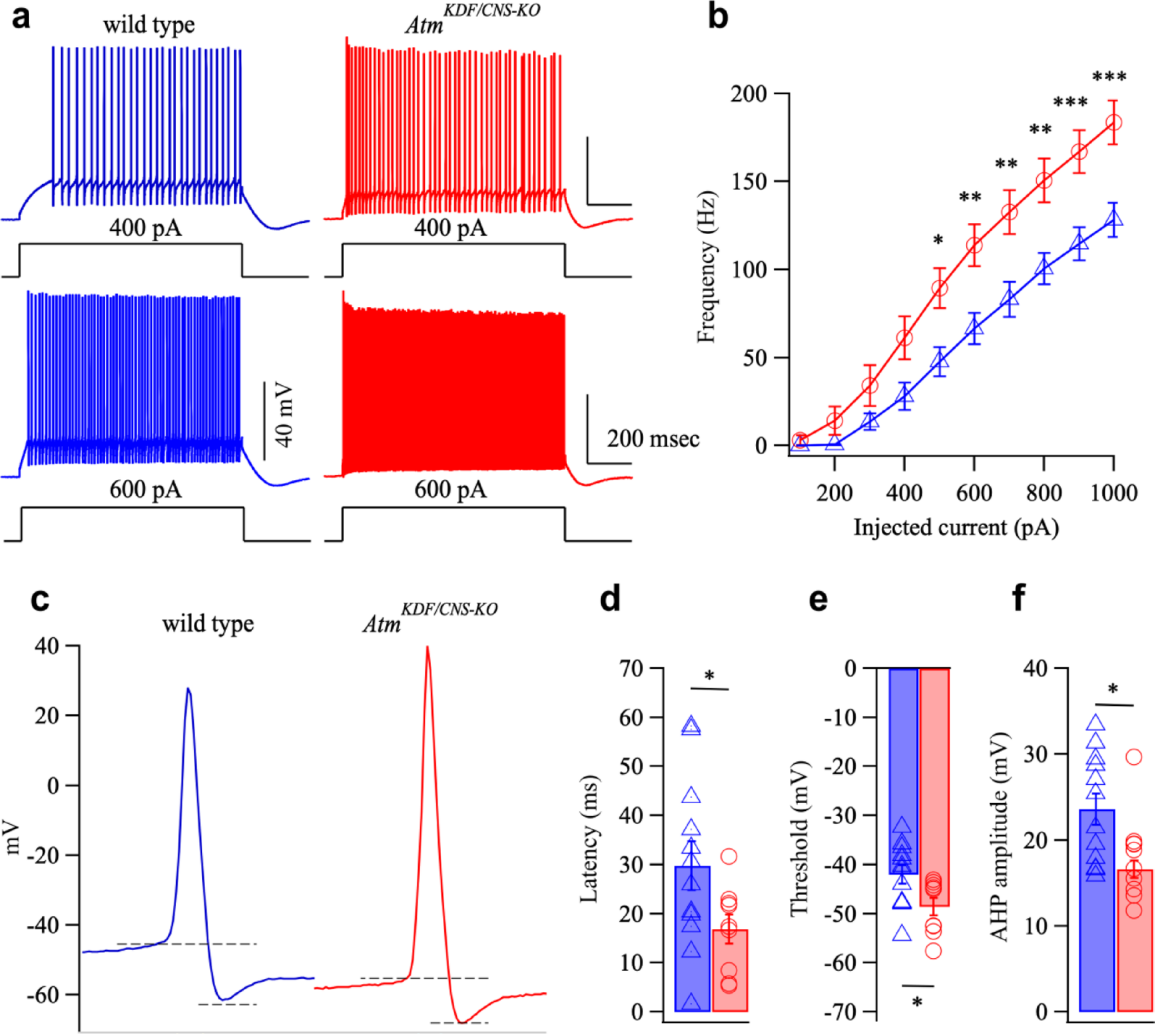
Increased intrinsic excitability of *Atm*^*KDF/CNS−KO*^ PCs. Representative traces of action potential firing evoked by injection of increasing depolarizing currents (**a**). Evoked firing frequency as a function of stimulation intensity (**b**) (*Atm*^*KDFCNS−KO*^: *n* = 13 cells; wild type: *n* = 10 cells). Representative action potential waveforms from wild type (blue) and *Atm*^*KDF/CNS−KO*^ PCs (red) (**c**). Mean latency (**d**) action potential threshold (**e**) and AHP (**f**) of wild type (*n* = 12 cells) and *Atm*^*KDFCNS−KO*^ (*n* = 9 cells) PCs. **p < 0.01, two way-ANOVA followed by Sidak’s *post hoc* analysis in (**c**); *p < 0.05, Student’s unpaired *t*-test in (**d-f**). Results are reported as mean ± SEM

To determine whether increased vulnerability of PCs was associated with altered intrinsic excitability, we performed electrophysiological recordings from PCs in acute cerebellar slices. Analysis of spontaneous firing recorded in cell-attached mode revealed comparable interspike interval and coefficient of variation between genotypes (Supplementary Fig. 4). In contrast, analysis of evoked firing, recorded in whole-cell current clamp configuration, revealed marked differences between *Atm*^*KDF/CNS−KO*^ and wild type PCs. We performed the analysis of evoked action potential discharge by applying a fixed depolarizing current step (600 pA) and found a significant shift to the left in the distribution of interspike intervals (*p* < 0.0001, K-S test) (Fig. 8a-c), an increase of the instantaneous frequency (*p* < 0.01, Unpaired Student’s t-test) without significant changes, between genotypes, in the coefficient of variation (*p* > 0.05, Unpaired Student’s t-test) (Fig. 8d). When PCs were challenged with progressively increasing depolarizing current steps, *Atm*^*KDF/CNS−KO*^ PCs displayed a significant higher number of action potentials compared to wild type mice across stimulus intensities (*p* < 0.01, Two-way ANOVA: genotype effect, F_(1, 21)_ = 10.34) (Fig. 9a, b). Consistent with a hyperexcitable phenotype, the latency to the first action potential was significantly shorter for *Atm*^*KDF/CNS−KO*^ PCs compared to wild type littermates (*p* < 0.05, Unpaired Student’s t-test: t_(17)_ = 2.22) (Fig. 9d). The analysis of the features of the first action potential (Fig. 9c) revealed a significant reduction of the threshold (*p* < 0.05, Unpaired Student’s t-test: t_(19)_ = 2.44) (Fig. 9e) and of the after hyperpolarization (AHP) (*p* < 0.01, Unpaired Student’s t-test: t_(19)_ = 3.04) (Fig. 9f). Overall, our analysis demonstrates that *Atm*^*KDF/CNS−KO*^ PCs present an early increase in intrinsic excitability compared to wild type mice.

### Early motor coordination impairment and gait disturbance in *Atm*^*KDF/CNS−KO*^ mice

**Fig. 10:**
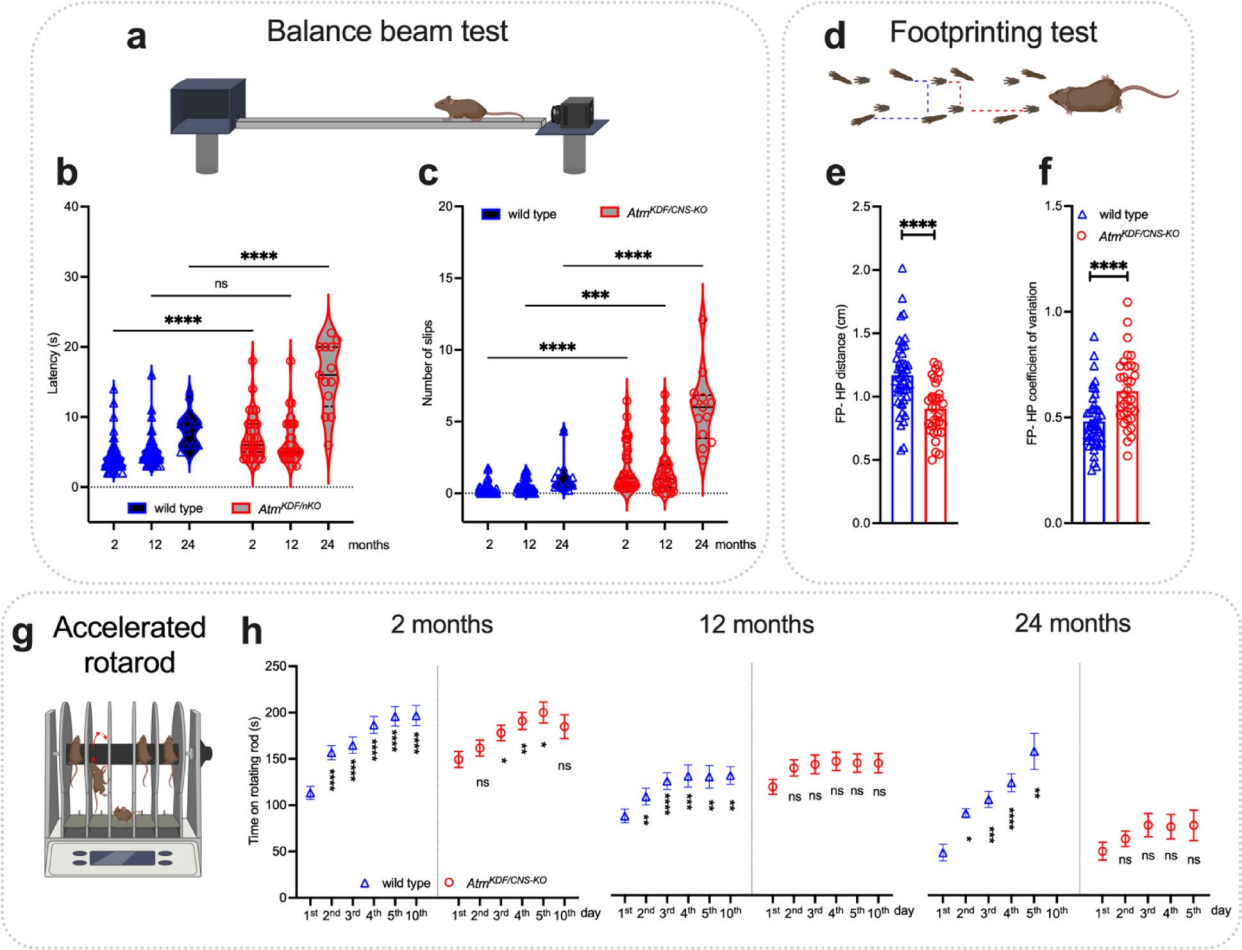
Impaired motor performance in *Atm*^*KDF/CNS−KO*^ mice. Motor coordination and gait were evaluated by means of balance beam test (**a-c**), rotarod test (**g-h**), and footprinting test (**d-f**) respectively. Schematic representation of balance beam test **(a).** 2 months old *Atm*^*KDF/CNS−KO*^ mice (*n* = 37) committed more errors than wild type littermates (*n* = 53) traversing the bar (**c**) and the time to cross the bar was higher for *Atm*^*KDF/CNS−KO*^ mice (**b**). *Atm*^*KDF/CNS−KO*^ mice (*n* = 29) of 12 months old continued to commit more errors than wild type (*n* = 40) and the latency was maintained higher (**b-c**). *Atm*^*KDF/CNS−KO*^ mice 24 months old have a worse phenotype. The latency and the number of errors is the highest over months (**b-c**). (***p < 0.001; ****p < 0.0001, Student’s t-test). Schematic representation of the footprinting test performed at 2 months of age in 2 months old mice of both genotypes **(d).** Significant reduction of the FP-HP distance in *Atm*^*KDF/CNS−KO*^ mice (*n* = 34) compared to wild type littermates (*n* = 45) (**e)**. Increased coefficient of variation of FP-HP in *Atm*^*KDF/CNS−KO*^ mice. (****p < 0.0001, Student’s t-test). Schematic representation of rotarod test **(g).** Time on rotating rod at 2 months (wild type mice: *n* = 43 and *Atm*^*KDF/CNS−KO*^ mice: *n* = 24; all comparisons *versus* 1st day, One way ANOVA RM); at 12 months (wild type mice: *n* = 30 and *Atm*^*KDF/CNS−KO*^ mice: *n* = 22; all comparisons *versus* 1st day, One way ANOVA RM); at 24 months (wild type mice: *n* = 7 and *Atm*^*KDF/CNS−KO*^ mice: *n* = 5; all comparisons *versus* 1st day, One way ANOVA RM). *p < 0.05; **p < 0.01; ***p < 0.001; ****p < 0.0001, Tukey’s Post hoc. Values are reported as mean ± SEM. Schematic representations were created using BioRender. https://BioRender.com/sgdt667

To determine whether the structural and functional cerebellar alterations at early stages translated into motor impairment, we assessed motor coordination and gait across disease progression, starting at 2 months of age, an early phase of the disease, and repeating the analysis at 12 and 24 months to monitoring the progressive worsening of the phenotype over time. The balance beam test is very effective in detecting very slight deficits in both motor coordination and balance (Fig. 10a). At 2 months of age, *Atm*^*KDF/CNS−KO*^ mice already displayed impaired motor performance in the balance beam task, characterized by increased crossing latency (*p* < 0.0001; Two-way ANOVA: genotype effect, F_(1, 114)_ = 61.49); for 2 months old mice: (wild type: 4.27 ± 0.27; *Atm*^*KDF/CNS−KO*^: 7.15 ± 0.55), for 12 months old: (wild type: 5.20 ± 0.39; *Atm*^*KDF/CNS−KO*^: 6.63 ± 0.60), and for 24 months old: (wild type: 8.10 ± 0.77; *Atm*^*KDF/CNS−KO*^: 15.77 ± 1.37) (Fig. 10b). The number of slips was higher compared to controls (*p* < 0.0001; Two-way ANOVA: genotype effect, F_(1, 185)_ = 134.4); for 2 months old mice (wild type: 0.25 ± 0.05; *Atm*^*KDF/CNS−KO*^: 1.80 ± 0.29), for 12 months old: (wild type: 0.46 ± 0.08; *Atm*^*KDF/CNS−KO*^: 1.59 ± 0.32) and for 24 months old: (wild type: 1.08 ± 0.35; *Atm*^*KDF/CNS−KO*^: 5.89 ± 0.70) (Fig. 10c). These deficits progressively worsened with age, indicating a gradual deterioration of motor coordination (genotype per age interaction effect: for latency, F_(2, 72)_ = 9.97; for number of slips, F_(2, 185)_ = 20.53).

We performed the footprinting test to analyze the gait characteristics in mice of both genotypes at 2 months of age. Our analysis indicated a significant reduction of the FP-HP placement (wild type: 1.17 ± 0.04 cm and *Atm*^*KDF/CNS−KO*^: 0.91 ± 0.04 cm; *p* < 0.0001, Unpaired Student’s t-test) as well as an increased coefficient of variation of FP-HP placement (wild type: 0.48 ± 0.02 and *Atm*^*KDF/CNS−KO*^: 0.62 ± 0.03; *p* < 0.0001, Unpaired Student’s t-test) (Fig. 10d-f). No significant changes between genotypes were observed for the other gait parameters and their coefficient of variation (for stride length: wild type: 5.9 ± 0.06 cm and *Atm*^*KDF/CNS−KO*^: 6.02 ± 0.09; for fore limb width: wild type: 3.40 ± 0.03 cm and *Atm*^*KDF/CNS−KO*^: 3.47 ± 0.05; for hind limb width: wild type: 3.71 ± 0.03 cm and *Atm*^*KDF/CNS−KO*^ : 3.67 ± 0.05 cm; for each gait parameter: *p* > 0.05, Unpaired Student’s t-test).

By means of accelerated rotarod test we evaluated the motor learning and memory in *Atm*^*KDF/CNS−KO*^ and wild type mice. Two-way ANOVA repeated measures revealed a significant time x genotype interaction effect (*P* < 0.05; F(5, 335) = 2.72) suggesting a different response of genotypes over time. Indeed, the learning over days of *Atm*^*KDF/CNS−KO*^ mice was delayed compared to wild type mice (2nd day vs. 1st day; one way ANOVA repeated measures, *P* > 0.05 Tukey’s *Post hoc*). Moreover, the 10th day *Atm*^*KDF/CNS−KO*^ mice did not have the retention of the memory (10th day vs. 1st day; one-way ANOVA repeated measures, *P* > 0.05 Tukey’s *Post hoc*) compared to wild type littermates (Fig. 10g, h). At 12 and 24 months of age *Atm*^*KDF/CNS−KO*^ mice could not learn the task over days compared to wild type littermates and they reached a plateau from day 2 to 10 (one way ANOVA repeated measures, *P* > 0.05) (Fig. 10h). This indicates a progressive deficit of motor learning in *Atm*^*KDF/CNS−KO*^ mice. The absolute time on the rotating rod is influenced by the body mouse weight while the motor learning is not dependent on it. We found a significant reduction in body weight of *Atm*^*KDF/CNS−KO*^ compared to wild type mice at each age point under analysis: for 2 months old mice: wild type (23.86 ± 0.40 g) and *Atm*^*KDF/CNS−KO*^ (20.10 ± 0.46 g); *p* < 0.0001, Mann-Whitney test), for 12 months old: wild type (37.67 ± 1.26 g) and *Atm*^*KDF/CNS−KO*^ (31.01 ± 1.40 g); *p* < 0.001, Unpaired Student’s t-test), and for 24 months old: wild type (34.34 ± 1.33 g) and *Atm*^*KDF/CNS−KO*^ (29.23 ± 1.73 g); *p* < 0.05, Unpaired Student’s t-test) which might explain the slightly higher time on the rotating rod.

## Discussion

In this study, we show that loss of Atm kinase activity in the CNS induces an early and coordinated remodeling of the cerebellar microenvironment without overt neuronal loss. By combining proteomic, structural, ultrastructural, electrophysiological and behavioral analysis, we demonstrate that Atm kinase deficiency is associated with ECM alterations, impaired white matter homeostasis and astrocytosis, and increased PC vulnerability. Together, these data indicate that cerebellar pathology in *Atm*^*KDF/CNS-KO*^ mice emerges from a destabilization of the tissue environment that compromises circuit integrity at early stages of the disease.

Our proteomic profiling revealed broad alteration across four major pathways in *Atm*^*KDF/CNS-KO*^ mice: *(i)* disorganization of the ECM, *(ii)* astrocyte modifications, (iii) downregulation of myelin/oligodendroglial proteins and *(iv)* alteration in neuronal excitability and synaptic organization. These findings indicate a global destabilization of cerebellar homeostasis rather than isolated molecular changes.

A central finding of our study is an early remodeling of the ECM in the cerebellar white matter of *Atm*^*KDF/CNS-KO*^ mice. The ECM remodeling is a well-recognized consequence of oxidative stress and DNA damage, hallmark features of A-T, through their effects on ECM synthesis, turnover, and stabilization [32]. The upregulation of the components of the basement membrane and the interstitial matrix suggests that ECM could be biochemically remodeled toward greater stiffness and rigidity. Such changes are known to aggravate oxidative stress and DNA damage [7, 32], stressors particularly detrimental in the absence of ATM. A properly regulated ECM is required throughout development and remains crucial in adulthood. It preserves network function by regulating cell survival, proliferation, migration and differentiation. Furthermore, it modulates synaptic plasticity and provides structural anchorage for both neuronal and glial processes [25].

Although ECM components are produced by both neurons and glial cells, astrocytes and microglia are the primary regulators of ECM composition and turnover [52]. In this context, our finding of increased in SOX9 + cells together with enhanced GFAP fluorescence, restricted to the white matter of *Atm*^*KDF/CNS-KO*^ mice, supports the presence of region-specific astrocytosis. This astroglial alterations may contribute to ECM remodeling in the white matter. Conversely, ECM alterations may themselves influence astrocyte state and function [25, 32]. This reciprocal influence suggests a self-reinforcing cycle in which mechanical and biochemical changes of the ECM and astroglial changes perpetuate each other, progressively destabilizing the cerebellar microenvironment.

The proteomic profile also revealed a coordinated downregulation of multiple myelin and oligodendrocyte proteins in the cerebellum of *Atm*^*KDF/CNS-KO*^ mice. Consistently, structural and ultrastructural analyses confirmed a marked reduction of myelination in both cerebellum and corpus callosum in *Atm*^*KDF/CNS-KO*^ mice. This occurred despite preserved axon and oligodendrocyte numbers, supporting the notion that *Atm*^*KDF/CNS-KO*^ mice display a defect in myelin maintenance rather than axonal or oligodendrocyte loss. Oxidative stress and DNA damage are known to impair the oligodendrocyte lineage maturation, and Atm deficiency may further exacerbate this vulnerability [54]. However, in our model, we did not observe evidence of impaired oligodendrocyte maturation. These findings suggest that the observed myelin deficits may not primarily reflect a failure of oligodendrocyte lineage progression, but rather arise in association with a profoundly remodeled and stiffened ECM and astrocytosis, two conditions that are known to create a non-permissive environment for the correct maintenance of myelin [25, 31, 52, 55].

The combination of stiffened ECM, astrogliosis and defective myelination in *Atm*^*KDF/CNS-KO*^ mice is associated with PCs exhibiting DCD and increased intrinsic excitability, indicating that these neurons are highly vulnerable in early stages of the disease. The influence of astrocytes, ECM and oligodendrocytes on neuronal excitability and synaptic function is well established [26, 49]. Moreover, the relationship between myelin and excitability is bidirectional: neuronal activity regulates myelination and myelin integrity modulates neuronal firing properties [37]. Thus, the altered excitability of PCs likely reflects the cumulative impact of microenvironment instability.

Although most ataxias are characterized by reduced PC excitability [16], the increased activity observed in *Atm*^*KDF/CNS-KO*^ mice likely represents an early and potentially transient compensatory response aimed at maintaining cerebellar output in the face of widespread microenvironmental disruption. Such compensation is likely unsustainable, ultimately contributing to circuit failure and motor impairment even in the absence of PC loss. Notably, reduced intrinsic excitability of PCs has been reported in a different murine model of A-T in later stages of the disease [39]. This discrepancy may be explained by differences in disease stage, suggesting that PC excitability undergoes dynamic changes during disease progression. The early motor impairment that we observed in our model is consistent with previous findings in other murine models of A-T, in which motor deficits emerge despite mild or absent cerebellar morphological abnormalities [4, 39], hence suggesting that functional disruption of cerebellar circuitry may represent a key determinant of the ataxic phenotype.

### Limitations of the study

In conclusion, although our data provide a comprehensive view of the molecular, structural, and functional alterations in the cerebellum of *Atm*^*KDF/CNS−KO*^ mice, they do not allow us to infer a temporal sequence or to identify a single primary causal event. Moreover, because the Nestin-Cre model targets multiple neural cell populations, our study cannot discriminate whether the observed ECM alterations arise from cell-autonomous mechanisms within specific cell types or from non-cell autonomous interactions among neurons, glial cells, and extracellular environment. Thus, the relationship between ECM remodeling, myelin defects, astrocytosis, and PC hyperexcitability should be interpreted as an association among interconnected pathological changes rather than as evidence of a linear pathogenic cascade.

Our findings support a coordinated disruption across multiple cellular compartments, both intra- and extracellular, suggesting that Atm kinase deficiency alters cerebellar homeostasis through a network level mechanism rather than a single pathway. The simultaneous upregulation of basement-membrane and interstitial ECM proteins, the downregulation of myelin-related proteins, the activation of astrocytic pathways and the increased excitability of PCs point to a multifaceted microenvironmental imbalance in which these processes likely interact bidirectionally. However, direct mechanistic links will require future studies using cell-type-specific genetic approaches or functional rescues experiments in *Atm*^*KDF/CNS−KO*^ mice. Therefore, while causality cannot be assigned to any single component, the convergence of these alterations is consistent with a model in which ATM contributes to the maintenance of cerebellar microenvironment homeostasis and highlights ECM-glia-neuron interaction as a potential therapeutic target in A-T.

## Supplementary Information

Below is the link to the electronic supplementary material.

Supplementary Material 1

Supplementary Material 5

## Data Availability

All data supporting the findings of this study are available from the corresponding author upon request.
