# Supplementary material for: ATM-kinase deficiency triggers early multi-compartment remodeling of the cerebellar microenvironment
Source: Acta Neuropathol Commun. 2026 May 29;14:161. doi: 10.1186/s40478-026-02332-9 (PMC13439767; doi:10.1186/s40478-026-02332-9)
Supplement: Supplementary file 1 [file 40478_2026_2332_MOESM2_ESM.docx]

**Supplementary Figure 1**

**Supplementary Fig. 1. Generation and validation of the Atm^KDF^ allele.** Schematic representation of the *Atm^KD^* and *Atm^KDF^* alleles and the predicted recombination products **(a)**. Targeting strategy for the Atm^KDF^ allele. GL, germline allele; KDFN, targeted allele prior to NeoR cassette excision. Restriction sites are indicated as follows: K, KpnI; RV, EcoRV; H3, HindIII. Exon numbers are labeled. The exon harboring the kinase-dead mutations is shown as a filled box. The 5′ and 3′ Southern blot probes are indicated by thick black lines **(b)**. Representative Southern blot analyses of correctly targeted ES cell clones. The expected sizes of germline (GL) and targeted alleles are indicated. Restriction enzyme digestions and probes used are shown below each blot **(c)**. Representative sequencing chromatograms confirming the introduced mutations. Corresponding genomic DNA and predicted protein sequences are aligned above **(d)**. The representative flow cytometry (FACS) analyses of lymphocyte development in VavCre+ Atm^KDF/C^ mice compared to *Atm^+/+^* and *Atm^-/-^* (null) mice (e).

**Supplementary Figure 2**

**Supplementary Fig. 2. *Atm^KDF/CNS-KO^* mice display preserved thickness of the molecular and granular layers of the cerebellum**. Schematic representation of cerebellar layers (**a**). Quantification of the thickness of the molecular (**b**) and granular layers (**c**) in the cerebellar slices from both genotypes (wild type n = 8; *Atm^KDF/CNS-KO^* n = 8). Data are represented as mean ± SEM. (p>0.05, Mann-Whitney test).

**Supplementary Figure 3**

**Supplementary Fig. 3. The oligodendrocyte cell density is maintained in *Atm^KDF/CNS-KO^* mice.** Oligodendrocyte lineage diagram showing that all cells of the oligodendrocyte lineage express the transcription factors SOX10 (red), OPCs also express the proteoglycan NG2 (white indicated by the arrow in the lower left images), and mature oligodendrocytes express the markers CC1 (white indicated by the arrow in the lower right images). Scale bar, 20 μm (**a**). Quantification of SOX10+ (**b**, **e**), SOX10+/NG2+ immature (**c**, **f**), and SOX10+/CC1+ mature (**d**, **g**) oligodentrocyte cell density in the white matter of cerebellum **(b-d**) and corpus callosum (**e-g**) from both genotypes (wild type n = 6; *Atm^KDF/CNS-KO^* n = 5 for cerebellum; and wild type n = 4; *Atm^KDF/CNS-KO^* n = 4 for corpus callosum). Data are represented as mean ± SEM. (p>0.05, Mann-Whitney test). Schematic representations were created using BioRender. https://BioRender.com/sgdt667.

**Supplementary Figure 4**

**Supplementary Fig. 4. Preserved Purkinje cell spontaneous firing in *Atm^KDF/CNS-KO^* mice.** Representative traces recorded in cell-attached configuration for wild type (blue) and *Atm^KDF/CNS-KO^* mice (red) **(a)**. Histograms represent the mean interspike interval (ISI) **(b)**, the ISI coefficient of variation (CV) **(c)** and the ISI CV2 (c) for wild type (*n* = 14) and *Atm^KDF/CNS-KO^* (*n* = 8) PCs. No significant differences were observed between genotypes for all the parameters analyzed (p > 0.05, unpaired Student's t-test). Data are represented as mean ± SEM.
